# Exploring the mechanisms behind autologous lipotransfer for radiation-induced fibrosis: A systematic review

**DOI:** 10.1371/journal.pone.0292013

**Published:** 2024-01-25

**Authors:** Nikhil Pattani, Jaspinder Sanghera, Benjamin J. Langridge, Marvin L. Frommer, Jeries Abu-Hanna, Peter Butler

**Affiliations:** 1 Epsom and St Helier NHS Trust, London, United Kingdom; 2 Walsall Healthcare NHS Trust, Birmingham, United Kingdom; 3 Department of Plastic Surgery, Royal Free Hospital, London, United Kingdom; 4 Division of Surgery & Interventional Sciences, University College London, London, United Kingdom; 5 Charles Wolfson Centre for Reconstructive Surgery, Royal Free Hospital, London, United Kingdom; 6 Division of Medical Sciences, University of Oxford, Oxford, United Kingdom; Affiliated Hospital of Jiangsu University, CHINA

## Abstract

**Aim:**

Radiation-induced fibrosis is a recognised consequence of radiotherapy, especially after multiple and prolonged dosing regimens. There is no definitive treatment for late-stage radiation-induced fibrosis, although the use of autologous fat transfer has shown promise. However, the exact mechanisms by which this improves radiation-induced fibrosis remain poorly understood. We aim to explore existing literature on the effects of autologous fat transfer on both in-vitro and in-vivo radiation-induced fibrosis models, and to collate potential mechanisms of action.

**Method:**

PubMed, Cochrane reviews and Scopus electronic databases from inception to May 2023 were searched. Our search strategy combined both free-text terms with Boolean operators, derived from synonyms of adipose tissue and radiation-induced fibrosis.

**Results:**

The search strategy produced 2909 articles. Of these, 90 underwent full-text review for eligibility, yielding 31 for final analysis. Nine conducted in-vitro experiments utilising a co-culture model, whilst 25 conducted in-vivo experiments. Interventions under autologous fat transfer included adipose-derived stem cells, stromal vascular function, whole fat and microfat. Notable findings include downregulation of fibroblast proliferation, collagen deposition, epithelial cell apoptosis, and proinflammatory processes. Autologous fat transfer suppressed hypoxia and pro-inflammatory interferon-γ signalling pathways, and tissue treated with adipose-derived stem cells stained strongly for anti-inflammatory M2 macrophages. Although largely proangiogenic initially, studies show varying effects on vascularisation. There is early evidence that adipose-derived stem cell subgroups may have different functional properties.

**Conclusion:**

Autologous fat transfer functions through pro-angiogenic, anti-fibrotic, immunomodulatory, and extracellular matrix remodelling properties. By characterising these mechanisms, relevant drug targets can be identified and used to further improve clinical outcomes in radiation-induced fibrosis. Further research should focus on adipose-derived stem cell sub-populations and augmentation techniques such as cell-assisted lipotransfer.

## Introduction

Radiotherapy serves as a cornerstone of cancer treatment. The procedure involves projecting high energy photons, or ionising radiation, towards a tumour to irreversibly damage the DNA of its malignant cells and prevent further neoplastic progression [1]. However, radiotherapy can indiscriminately damage surrounding healthy tissue leading to unwanted side effects [2]. With the growing number of cancer survivors due to general advances in cancer treatment, clinicians increasingly deal with these dreadful side effects.

Radiation-induced fibrosis (RIF) is a recognised consequence of external beam radiotherapy, especially after multiple, prolonged dosing regimens. This can be defined as the overgrowth, hardening, and/or scarring of the skin, underlying soft tissue and/or organs to which radiation has been applied. Its pathophysiology is characterised by acute injury followed by misguided healing, involving excessive deposition of extracellular matrix (ECM) components such as collagen [2]. This reaction can occur in several areas, including but not limited to the skin, subcutaneous tissue, lungs, breasts and gastrointestinal tract.

Radiation-induced skin fibrosis is commonly seen, since the skin and subcutaneous tissue overlying the target tumours are particularly radiosensitive. This is due to the high proliferative capacity and oxygenation requirements of the basal epidermal cells [3]. Over 90% of patients receiving radiotherapy develop moderate to severe skin reactions, on a spectrum from acute radiodermatitis to chronic RIF [4]. The impact of this condition on tissue form and function can be immense and significantly worsen quality of life. For example, head and neck cancer patients may experience both cosmetic and functional impairment with skin tightening and thickening, trismus, neck pain and cervical dystonia in combination with volumetric defects [5].

Immediately post-radiotherapy, patients may experience acute dermatological signs such as mucositis, rashes, and erythema [1]. Instead, RIF of the skin is a chronic complication post-radiotherapy with its onset often being delayed by six months to several years. Initial warning signs may include pain, ulceration, and induration of the affected site, which may resolve without intervention [6]. As it progresses, features such as hypo-/hyper-pigmentation, skin atrophy, and loss of hair follicles, nails and sebaceous glands may present [7]. In severe late-stage RIF of the skin, the underlying subcutaneous tissue is affected resulting in contractures, necrosis and lymphoedema [8].

The pathogenesis of RIF of the skin is complex involving several different inflammatory and immunological mechanisms across a long timespan. Radiation injury results primarily from the production of reactive oxygen species, which damage surrounding cellular materials [9]. Cellular injury results in the release of chemoattractant molecules causing an acute inflammatory response, with fibroblast migration and ECM deposition [9–11]. Immune cells such as neutrophils are attracted to the site of injury and release further pro-inflammatory cytokines [12]. Later on, lymphocytes arrive and macrophages differentiate into their sub-types (M1 and M2) [13]. This eventually leads to the generation of pro- fibrotic molecules such as transforming growth factor beta (TGF-β), which results in fibroblast production and their subsequent differentiation into myofibroblasts. Myofibroblasts secrete excessive amounts of collagen and fibronectin, resulting in the late effects of RIF such as thickening, contractures etc. [14].

To date, there is no definitive treatment for late-stage RIF of the skin. For decades, it was considered an inevitable, progressive, and irreversible condition which must be tolerated in order to achieve complete cancer eradication. However, the use of autologous fat transfer (AFT), or autologous lipotransfer, has shown promise as treatment for RIF. Adipose tissue can easily be harvested in most patients [15]. From the first AFT procedure performed by Rigotti *et al*. in 2007, the field has expanded with multiple variations in graft content and surgical techniques [16]. Later, it was observed that fat grafting not only served as a filler, but also enhanced the quality of the surrounding tissue, seemingly making overlying scars fade. A study by Griffin *et al*. observed aesthetic and functional improvements in RIF of the skin in 97% of their patients treated with AFT [17]. It was further found to significantly improve psychological health. Histological research demonstrated a reduction, remodelling, and realignment of collagen fibres, increased vascularization, and reduction of contractile proteins, dermal thickness, and scar size [18,19].

To date, the exact mechanisms by which AFTs improve fibrosis remain poorly understood. Fat tissue mainly consists of adipocytes, but a variety of other cells are also present collectively named stromal vascular cells. The stromal vascular fraction (SVF) consists of a mix of cells including immune cells, endothelial cells, smooth muscle cells, pericytes, preadipocytes and adipose derived stem/stromal cells (ADSCs). Although only accounting for a small percentage of the cells present in lipotransfers, ADSCs are believed to be of central importance in the grafts anti-fibrotic effects. This is believed to be due to their inherent regenerative capacity, but especially their paracrine signalling. ADSCs are common to all lipotransfer techniques (including whole adipose grafts, stromal vascular fraction, cell-assisted lipotransfer (CAL) etc.).

In this review, we aim to explore existing literature on the use of AFT in both in-vitro and in-vivo RIF skin models, and to collate potential mechanisms by which this procedure takes effect against the condition.

## Methods

A protocol was created according to the Preferred Reporting Items for Systematic Review and Meta-Analyses Protocols (PRISMA-P) statement [20]. The search strategy as follows was designed by BL, JAH, JS, and NP.

### Information sources

PubMed, Cochrane reviews and Scopus electronic databases from inception to May 2023 were searched. Our search strategy for PubMed combined both free-text terms and Medical Subject Headings (MeSH) with Boolean operators, as shown in Table 1. The search strategies for the latter two databases consists of free-text terms and Boolean operators only. These are shown in Table 2.

**Table 1:** PubMed search strategy.

Search string No.	MeSH headings	Free-text terms
**1**	adipose tissue OR mesenchymal stem cells OR stromal vascular function OR stromal cells	lipoinject* OR fat inject* OR fat trans* OR Fat aspirate OR lipoaspirate OR lipotrans* OR fat graft* OR mesenchymal stem cell* OR adipose-derived stem cell* OR adipose derived stem cell OR fat transf* OR lipofill* OR lipomodell* OR adipose graft* OR adipose stem cell* OR adipose transplant* OR ASC OR ADSC* OR MSC OR SVF OR stromal cell OR adipose regenerative cell OR stromal vascular fraction OR adipocyte progenitor OR pre-adipocyte
**2**	radiation fibrosis OR radiodermatitis OR radiodermatitides OR high energy radiotherapy OR ionizing radiation OR radiation fibrosis syndrome OR radiation induced dermatitis	radiotherapy-induced fibrosis OR ionising radiation OR ionizing radiation OR radiotherapy fibrosis OR radiation-induced fibrosis OR cutaneous radiation syndrome

**Table 2:** Scopus and Cochrane reviews.

Search string No.	Free-text terms
**1**	Adipose tissue OR Lipoinject* OR fat inject* OR fat trans* OR Fat aspirate OR Lipoaspirate OR Lipotrans* OR fat graft* OR mesenchymal stem cell* OR Stromal cell* OR adipose-derived stem cell* OR adipose derived stem cell OR fat transf* OR lipofill* OR lipomodell* OR adipose graft* OR adipose stem cell* OR adipose transplant* OR ASC OR ADSC* OR MSC OR SVF OR adipose stromal cell OR adipose regenerative cell OR stromal vascular fraction OR adipocyte progenitor OR Pre-adipocyte
**2**	Radiation fibrosis OR radiodermatitis OR radiodermatitides OR High energy radiotherapy OR Radiotherapy-induced fibrosis OR ionising radiation OR ionizing radiation OR radiotherapy fibrosis OR radiation-induced fibrosis OR cutaneous radiation syndrome OR Ionizing radiation OR Radiation fibrosis syndrome OR radiation induced dermatitis

### Study selection

**Fig 1:**
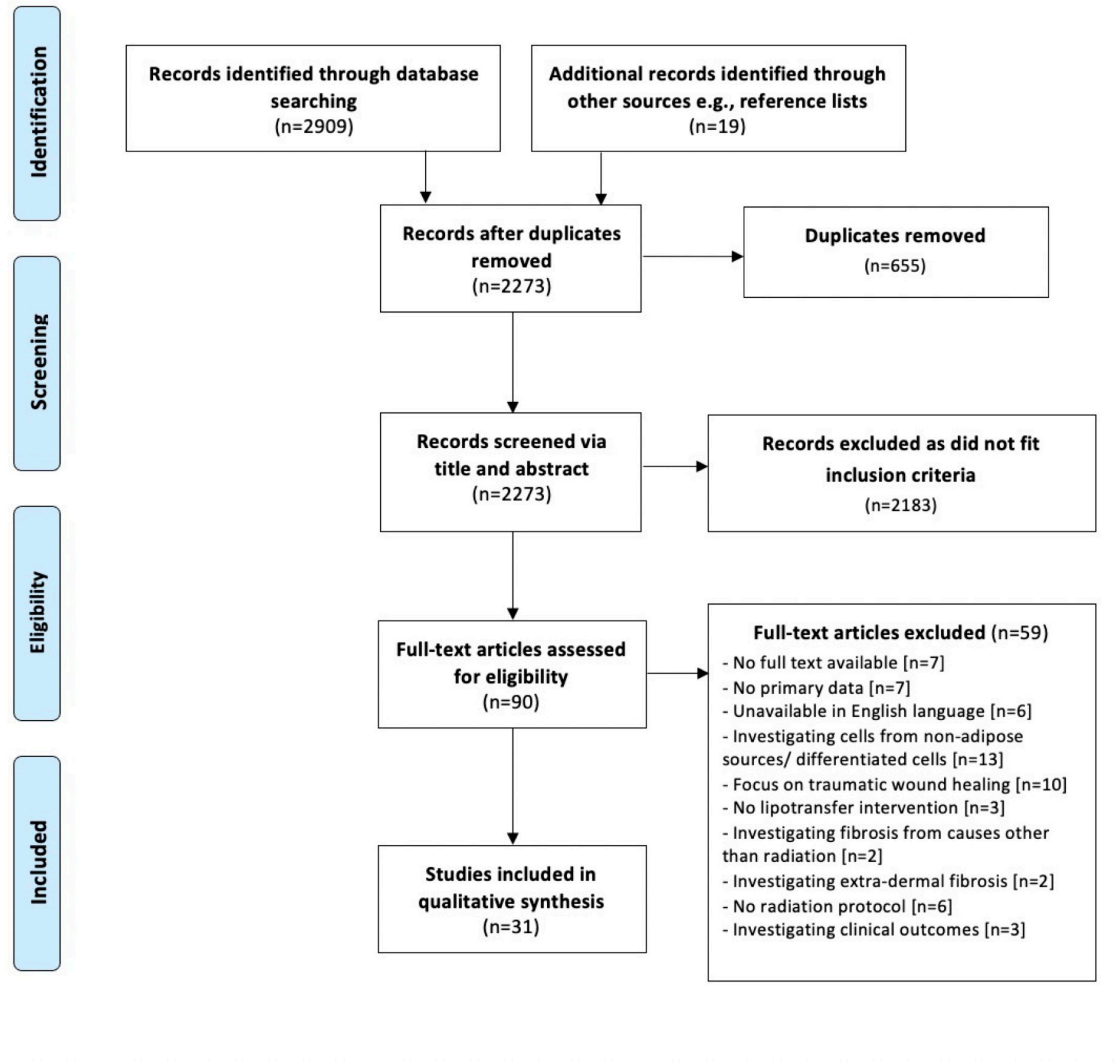
PRISMA flowchart.

Two authors (NP and JS) screened the compiled list of search results. These were managed using Endnote 20 (Thomas Reuters, Toronto, Canada) and Excel (Microsoft, Redmond, Washington, USA). After removing duplicates, the articles were independently screened by two authors (NP and JS) in parallel, first by title and abstract. Any disagreements were reviewed by the third and fourth author (BL and JAH). Those remaining were studied in full, prior to a final shortlisting based on pre-specified inclusion/exclusion criteria listed below. To ensure all relevant studies were identified, the reference lists of all included articles and previous systematic reviews were routinely checked. This process is displayed diagrammatically as a PRISMA flowchart in Fig 1.

### Eligibility criteria

Inclusion criteria:

1. Studies utilising photon/electron radiation
2. Studies utilising adipose tissue fractions or single cell groups isolated from adipose tissue as their intervention
3. In vitro/in vivo studies with primary data
4. Studies published in English language

Exclusion criteria:

1. Studies solely focused on fibrosis of visceral organs
2. Clinical trials
3. Review papers and other non-primary data sources
4. Studies focusing on differentiated cells or stem cells derived from non-adipose sources e.g., bone marrow- derived mesenchymal stem cells

### Selected articles

The search was performed on 13^th^ May 2023. The search strategy (Tables 1 and 2) yielded 2909 studies initially, and then 2273 after duplicates removed. After title and abstract screening, 90 articles underwent full-text review for eligibility. Of these articles, 59 were excluded in-line with our inclusion/exclusion criteria. In total, 31 papers underwent analysis in the final systematic review.

### Data collection process

All data from the final studies was extracted onto an electronic proforma using Excel (Microsoft, Redmond, Washington, USA) by two authors (NP and JS). This proforma was pre-designed by BL and trialled on three articles. It was deemed to provide a consistent and structured method of data extraction and appraisal. Any disagreements with extracted data were rectified by BL and JAH.

For studies which provided information on multiple interventions, skin conditions and/or research outcomes, only data relating to the current research objective was extracted. Where appropriate, authors were contacted to provide missing information.

### Data items

Data items extracted included study design (in vivo/in vitro), intervention preparation, cell types used, disease model, radiation protocol e.g., dosage regimens (Grays, fractionation), date of last follow-up and recorded outcomes. Where mechanisms behind AFT in RIF of the skin were suggested in studies, this and corresponding evidence were also collated.

### Risk of bias assessment

All included studies involved a non-randomised design comparing both effector/intervention and comparator/control groups. Without randomisation these observational results may be inherently biased. The risk of bias for *in-vivo* studies was assessed using the Systematic Review Centre for Laboratory Animal Experimentation (SYRCLE) assessment tool [21]. The Office of Health Assessment and Translation (OHAT) tool designed by the United States national toxicology program was used for *in-vitro* studies [22].

Two authors (NP and JS) independently appraised each study, assigned individual scores for each tool domain, and then presented this information in risk of bias tables. These are shown in S1 File. Any discrepancies between authors were resolved by consensus with authors BL and JAH.

### Data synthesis

As there is no standardised protocol or unit of analysis for assessing the effects of AFT in RIF, methodologies vary between studies. An overarching meta-analysis and quantitative synthesis was not conducted on account of this between-study heterogeneity. Instead, outcome measures were evaluated using simple descriptive statistics and results collated in a narrative synthesis. The results were split into *in vivo* and *in vitro* experiments and separated further by effector cell (fibroblast, immune cells, vascular endothelial cells (VEC) etc.). The effect of AFTs on vascular function, immunomodulation and fibrosis were summarised in the review.

## Results

### Literature search summary

In total, 31 studies were included for final analysis. Of these, nine conducted *in vitro* experiments utilising a co-culture model whilst 25 studies conducted *in vivo* experiments. The results are summarised below in *Tables 3* and *4*.

**Table 3:** *In-vitro* studies.

Paper	Intervention preparation	ADSC characterisation	Other cell types	Disease model	Radiation protocol	Outcomes	End- point(s)	Results	Conclusions
**Haubner et al. [23]**	Collagenase digestion, filtration, and centrifugation of human adipose tissue ADSCs cultured to passage 5/6	Details not provided	HDMEC	Intervention: HDMEC/ADSC direct co-culture in endothelial cell growth medium (irradiated) Controls: 1.HDMEC/ medium (irradiated); 2.ADSC/medium (irradiated); 3.HDMEC/ADSC (non-irradiated)	Single exposure of 2, 6 and 12Gy	Cell viability (Cedex XS Analyzer System) Cytokine production (ELISA: ICAM, VCAM-1, IL-6, FGF)	48h post-radiation 48h post-radiation	ICAM-1, VCAM-1, IL-6 and FGF secretion significantly lower in HDMEC/ADSC co-culture compared to controls	Radiation-induced endothelial cell dysfunction may be mitigated by ADSCs through decreased expression of pro-atherogenic adhesion molecules
**Shukla et al. [24]**	Collagenase digestion, filtration, and centrifugation of human adipose tissue ADSC-CM collected after 72h of culture	Not applicable	NDHF	Intervention: ADSC-CM/NHDF (irradiated) Controls: 1.NDHF alone (non-irradiated); 2.NDHF/control media (irradiated)	Single exposure of 10Gy	Gap closure (scratch migration assay)	48h post-radiation	17.5% reduction in NHDF migration after ADSC-CM treatment	ADSC-CM reversed dysregulated NHDF hypermigration post-radiation
**Haubner et al. [25]**	Collagenase digestion, filtration, and centrifugation of human adipose tissue ADSCs cultured to passage 5/6	Details not provided	NHDF	Intervention: NHDF/ADSC direct co-culture in fibroblast growth medium (irradiated) Controls: 1.ADSC alone (irradiated); 2.NHDF alone (irradiated); 3.NHDF/ADSC (non-irradiated)	Single exposure of 2, 6 and 12Gy	Cell proliferation (BrdU colorimetric assay) Gene expression/proteomic profile (RT-qPCR: MMP1, MMP2, MMP13; ELISA: TGF-β1, TIMP1 and TIMP2)	48h post-radiation 48h post-radiation	Increased MMP1 and MMP2 expression in irradiated NHDF/ADSC co-culture Increased secretion of TIMP1 and TIMP2 in irradiated NHDF/ADSC co-culture	ADSC-induced increased MMP1 expression may stimulate neovascularization and fibroblast migration The balance of MMPs and TIMPs may be altered by ADSCs to regulate repair
**Yao et al. [26]**	Collagenase digestion, filtration, and centrifugation of rat adipose tissue ADSCs cultured to passage 3 and supernatant derived	Flow cytometry (CD10, CD34, CD45, CD73, CD90, and CD105)	HaCat NOK	Intervention: HaCaT/NOK/ADSC culture- supernatant (irradiated) Control: HaCaT/NOK/normal culture medium (irradiated)	Single exposure of 5 and 20Gy	Cell apoptosis (TUNEL assay) Proteomic profile (Western Blotting: CTSF, Bid, BAX and Caspase-9)	72h post-radiation 72h post-radiation	Lower cell apoptosis rates and downregulated CTSF, BAX, Bid, and Caspase-9 secretion in the culture supernatant-treated group	ADSC-culture supernatant markedly attenuated radiation-induced apoptosis by downregulating CTSF and downstream pro-apoptotic proteins, and upregulating anti-apoptotic proteins.
**Ejaz et al. [27]**	Collagenase digestion, filtration, and centrifugation of human subcutaneous white adipose tissue ADSCs cultured to passage 4	Details not provided	HFF	Intervention: transwell HFF/ADSC co-culture (irradiated, ADSCs added 24h post-irradiation and co-culture maintained for 48h) Controls: 1.HFF (non-irradiated); 2.HFF/ADSC (non-irradiated)	Single exposure of 10Gy	Fibrosis-associated gene expression (RT-qPCR: TGF-β1, CTGF, NK-kB, IL-1, Col1-Col6, TNF and HGF) Proteomic profile (Luminex assay and western blotting: HGF)	72h post-radiation 72h post-radiation	Downregulation in the expression of fibrosis- associated genes and increased HGF production in irradiated HFF/ADSC co-culture	HGF production by ADSCs may mediate anti-fibrotic effects through down-regulation of fibrosis associated genes and bone marrow cell migration
**Xiao et al. [28]**	Collagenase digestion, filtration, and centrifugation of human subcutaneous white adipose tissue Mature adipocytes collected	Not applicable	HaCaT WS1	Intervention: transwell mature adipocyte/HaCaT/WS1 co-culture (irradiated) Controls: 1.WS1 (irradiated); 2.HaCaT (irradiated) Sub-experiments: 1.HaCaT incubated with fresh medium containing EGFP-tagged FABP4 protein; 2.PA incubated with WS1	Single exposure of 5 and 20Gy	Cell migration assay Cell proliferation assay Cell cycle analysis	24h post-radiation 24h post-radiation 24h post-radiation	Adipocytes increased fibroblast migration, but did not increase proliferation of co-cultured HaCat and WS1 FABP4 reduced radiation-induced DNA damage, whilst PA independently promoted migration of irradiated cells	Adipocytes facilitate the migration and repair of irradiated fibroblasts possibly through fatty acids (PA) and FABP4
**Saijo et al. [29]**	Source of ADSCs not provided ADSCs cultured to passage 4–6, with ADSC-CM obtained after culture	Details not provided	HDLEC	Interventions: HDLEC/ADSC and HDLEC/ADSC/ADSC-CM co-cultures (irradiated) Controls: 1.HDLEC monoculture (irradiated); 2.ADSC-CM (irradiated); 3.HDLEC/ADSC-CM (non-irradiated)	Single exposure of 1, 2, 6, or 12Gy	Cell proliferation (DAPI assay) Cell migration (scratch assay) Tube formation assay Proteomic profile (ELISA: bFGF, HGF, IGF-1, VEGF-A, VEGF-C, and Western Blotting: bFGF, VEGF-A, VEGF-C)	120h post-radiation 12h post-radiation 12h post-radiation 168h post-radiation	ADSCs reversed radiation-induced suppression of HDLEC proliferation, potentially through lymphangiogenic factor upregulation (bFGF in particular) Addition of ADSC-CM resulted in significantly smaller scratches and approximately two-fold longer tubes	ADSC-CM and ADSCs show similar effects on the lymphangiogenic ability of HDLEC (increased proliferation, migration, and tube formation)
**Yu et al. [30]**	Collagenase digestion, filtration, and centrifugation of human white adipose tissue Supernatants and adipocytes discarded, and SVF obtained	Flow cytometry (CD29, CD11b, CD73, CD45, CD105, CD90, CD34, HLA-D)	WS1	Intervention: WS1/SVF transwell co-culture (irradiated) Control: WS1 monoculture (irradiated)	Single exposure of 5Gy	Proteomic profile (TMT based proteomic quantification) Further GO annotation of proteome and KEGG pathway analysis	48h post-radiation	Upregulated proteins included DMTF1, MFAP5, PI16, MMP-1 and collagen chains KEGG-based pathway enrichment analysis significant upregulation of ECM-receptor interaction and focal adhesion pathways	SVF altered the fibroblast proteomic profile, further research is required to investigate significance.
**Sörgel et al. [31]**	Immortalised ADSCs (details not provided) enriched in cell medium ADSC-CM derived through centrifugation	Not applicable	Keratino-cytes	Interventions (irradiated 2D transwell co-culture and 3D matrix): 1.ADSC-CM/keratinocytes 2.IGF-1/keratinocytes 3.KGF/keratinocytes 4.platelet-lysate (PL)/keratinocytes 5.hylauronic acid (HA)/keratinocytes Control: keratinocyte monoculture (non-irradiated)	2 or 5Gy	Cell viability (WST-8 assay) Cell migration (Oris^™^ assay) Gene expression/ (qRT-PCR: p53, TNF, TGF-β1, IL-6, IL-8, IL-1β, COL1A1)	1, 4, and 7 days post-radiation 4, 12, and 20h post-radiation 25h post-radiation	Improvement in cell viability, migration and TNF/IL-6 expression in HA and IGF-1 groups Nil improvement in cell viability/ migration ADSC-CM and PL groups	ADSC-CM had no impact on irradiated keratinocyte models Hyaluronic acid and IGF-1 may aid in alleviating radiation-induced fibrosis

Key.

ADSCs- adipose-derived stem cells.

ADSC-CM- adipose-derived stem cell conditioned media.

BAX—Bcl-2-associated X *protein*.

Bid- BH3-interacting domain death agonist.

CASPASE- cysteine-aspartic proteases, cysteine aspartases.

Col1-Col6- collagens 1–6.

CSTF- cleavage stimulation factor.

CTGF- connective tissue growth factor.

DMTF1- cyclin-D-binding Myb- like transcription factor 1.

FABP4- fatty Acid Binding Protein 4.

FGF–fibroblast growth factor.

FGF2- basic fibroblast growth factor.

GO- Gene Ontology.

HaCaT- human immortalized keratinocytes.

hbFGF- human basic fibroblast growth factor.

HDLEC- human dermal lymphatic endothelial cells.

HFF- human foreskin fibroblasts.

HGF- hepatocyte growth factor.

HMDEC- human dermal microvascular endothelial cells.

ICAM-1- Intercellular Adhesion Molecule 1.

IL-1- interleukin-1.

IL-6- interleukin 6.

KEGG-Kyoto Encyclopaedia of Genes and Genomes.

KGF—keratinocyte growth factor.

mBMSC—mouse bone marrow stromal cells.

MFAP5- Microfibrillar associated protein 5.

MMP1- matrix metalloproteinase-1.

MMP2- matrix metalloproteinase-2.

MMP13- matrix metalloproteinase-13.

NK-kb- nuclear factor kappa-light-chain-enhancer of activated B cells.

NHDF- normal human dermal fibroblasts.

NOK- normal human oral squamous epithelial cells.

PA- palmitic acid.

PI- peptidase inhibitor.

SVF- stromal vascular fraction.

TGF-B—transforming growth factor beta.

TIMP1- TIMP Metallopeptidase Inhibitor 1.

TIMP2- TIMP Metallopeptidase Inhibitor 2.

TNF-α - tumour necrosis factor alpha.

VCAM-1- Vascular cell adhesion protein *1*.

WS1- human skin fibroblast cells.

**Table 4:** *In-vivo* studies.

Paper	Intervention preparation	ADSC characterisation	Disease model	Radiation protocol	Outcomes	Endpoint(s)	Results	Conclusions
**Borrelli et al. [32]**	Collagenase digestion, filtration and centrifugation of human adipose tissue ADSCs cultured to passage 3	FACS (CD34+, CD74+, CD74-)	Mice (n = 20) grafted 4wk post-radiation Interventions (ADSCs): 1.CD74+; 2.CD74−; 3.unsorted Control: lipoaspirate only	30Gy fractionated into six 5Gy doses on alternate days (total = 12 days)	Fat retention (Micro CT) Mechanical strength testing (Microtester) Histology (H&E) IHC (versican, elastin and fibrillin) FACS (fibroblast subpopulations)	8 wk post-graft	CD74+ ADSC enriched group showed greater integrity, reduced inflammation and fibrosis, decreased collagen and versican staining, and decreased proportion of fibrotic papillary and reticular fibroblasts, but significantly increased proportions of more anti-fibrotic zigzag and lipofibroblasts	Fat grafts enriched with CD74+ ADSCs showed improved histological quality, decreased pro-fibrotic protein production, and influenced fibroblast sub-populations
**Sultan et al. [33]**	Collagenase digestion, filtration and centrifugation of human adipose tissue Whole fat obtained	Not applicable	Mice (n = 25) Interventions: 1.Irradiated/whole fat graft; 2.Irradiated/sham graft (saline) Control: non-irradiated	45Gy single exposure	Histology (H&E) IHC (CD31 and Smad3) Scar index (Picrosirius red staining for collagen)	4 and 8 wk post-graft	Fat-grafted mice showed reduced hyperpigmentation, ulceration, epidermal thickness (2.6x), scar index, CD31 and Smad3 (pro-fibrotic marker) staining compared to sham- and control groups	Fat grafting slows fibrosis progression perhaps by reducing microvascular damage, but no mechanistic conclusions can be derived
**Forcher-on et al. [34]**	Collagenase digestion, filtration and centrifugation of minipig adipose tissue ADSCs cultured to passage 3–8	Flow cytometry (CD90, CD44, CD45, and CD31)	Minipigs (n = 13) grafted post-radiation Intervention: ADSC graft (50x10^6^ cells x4 over 3mo) Control: PBS injection	51Gy single exposure	Clinical score (pathological signs and pain evaluation) IHC (vWF and cytokeratin) Histology (H&E) ADSC movement (Q-dot and labelling)	140 days post-graft	Intervention group showed complete epidermal recovery (cytokeratin), and earlier lymphocyte infiltration into the dermis with greater vascularization (vWF) Grafted ADSCs were localised at dermis/subcutis barrier but not the epidermis	Owing to lymphocyte co-localization, ADSCs could potentially attract immune cells via paracrine mechanisms No evidence of ADSC differentiation into keratinocytes
**Riccob-ono et al.** **[35]**	Collagenase digestion, filtration and centrifugation of minipig adipose tissue (autologous) and two other animals (allogeneic) ADSCs cultured to passage 2	Flow cytometry (CD90, CD44, CD45, and CD31)	Minipigs (n = 18) grafted post-radiation Interventions: 1.autologous ADSCs; 2.allogeneic ADSCs (50x10^6^ cells x4 over 3mo) Control: PBS injection	50.6Gy ± 4.1Gy single exposure	Clinical score (pathological signs and pain evaluation)	119–124 days post-radiation	Final clinical score of autologous ADSC-grafted intervention group significantly improved compared to control group (less erythema, induration, oedema etc.) No significant difference in clinical score between allogenic ADSC intervention group and control group	Autologous ADSCs improved healing of radiation wounds, but reason for failure of allogeneic ADSCs is unclear Inflammation profile of both ADSC subtypes should be explored further
**Garza et al.** **[36]**	Collagenase digestion, filtration and centrifugation of human adipose tissue Whole fat obtained	Not applicable	Mice (n = 9) Intervention: irradiated/whole fat graft Control: non-irradiated/whole fat graft	30 Gy fractionated into 6 fractions of 5Gy (total = 12 days)	Collagen staining (Picrosirius red) IHC (CD31) Histology (H&E)	8wk post-graft	Lower levels of collagen and increased vascular density (CD31) in fat-grafted irradiated tissue compared to pre-graft irradiated tissue At 2wk, irradiated mice demonstrated significant dermal thinning compared to control, extending to 8wk Lower graft retention in irradiated group, but similar quality of remaining fat to control	AFTs may contribute to improved skin quality through neoangiogenesis, reduced collagen deposition and reduced dermal thickness
**Riccob-ono et al. [37]**	Collagenase digestion, filtration and centrifugation of minipig adipose tissue ADSCs grown to 85–95% confluence, proportion transfected with Shh plasmid	Flow cytometry (no markers provided)	Minipigs (n = 14) grafted post-radiation Interventions: 1.autologous ADSC; 2.ShhADSC (50x10^6^ cells x4 over 3mo) Control: PBS-injection	50.6 ± 4.1Gy single exposure	Clinical score (pathological signs and pain evaluation) *Autologous ADSC and PBS control groups are historical results	55–92 days post-radiation	Shh-ADSC injections well-tolerated, with clinical benefit (skin repair and pain decrease) similar to autologous ADSC group	Shh gene therapy initially hypothesised to enrich secretome and improve cell proliferation and neoangiogenesis, but no added clinical benefit seen
**Luan et al. [38]**	Collagenase digestion, filtration and centrifugation of human adipose tissue ADSCs harvested for CAL, and fat centrifuged for grafting	No details provided	Mice (n = unknown) Interventions: 1.CAL/irradiated; 2.Fat graft/irradiated Controls: 1.CAL/non-irradiated; 2.Fat graft/non-irradiated	30Gy fractionated into 6 doses of 5 Gy, (total = 12 days)	Fat graft retention volume (micro CT) IHC (CD31) Histology (H&E) Tensile stress strength (Force transducer)	8wk post-graft	The CAL/irradiated group showed significantly reduced dermal thickness and collagen content, and improved skin vascularity and stiffness in comparison to the fat/irradiated group Measures were comparable to non-irradiated controls	CAL-treated mice showed improved pathological markers, providing evidence for ADSC enrichment of fat grafts
**Ejaz et al. [27]**	Collagenase digestion, filtration and centrifugation of GFP+/Luc+ mice adipose tissue ADSCs cultured to passage 4	No details provided	Mice (n = unknown) grafted post-radiation Intervention: ADSCs (injected into hind limbs) Control: saline injection	35Gy single exposure	Leg movement (protractor) Collagen staining (Masson’s trichrome) Histology (H&E) ADSC tracking (GFP+/Luc+ expression)	28 days post-radiation 50 days post-radiation 77 days post-radiation	ADSC-treated mice showed improved limb movement, and reduced epithelial thickness and collagen density compared to control Positive luciferase signals seen at day 77 post-ADSC injection	ADSCs injected at irradiated sites persist and coupled with the in-vitro results of increased HGF production, suggest a local paracrine mechanism
**Lindeg-ren et al. [39]**	Whole fat obtained from breasts of female patients with treated breast cancer (surgery + adjuvant radiotherapy)	Not applicable	Female patients (n = 30) Intervention: whole fat/irradiated Control: non-irradiated/nil graft (contralateral breast of same patients)	Adjuvant radiotherapy given at least 12 months prior to patient recruitment (dose not provided)	Gene expression analysis (microarrays) IHC (CD86)	12mo post-graft	19.2% of the 3000 genes evaluated had significantly less variation in expression between the respective sides after AFT than before AFT Macrophage ratio (CD86) in irradiated vs. non-irradiated breast lower after AFT	AFT suppresses pathways involved in inflammation (IFN-γ response), fibrosis and hypoxia even a year post-radiation exposure
**Bertran-d et al. [40]**	Human adipose tissue grafts from healthy 35-year-old female, (Microfat (MF) + SVF isolated through collagenase digestion, PRP from blood sample)	Not applicable	Mice (n = 45) Interventions (irradiated): 1.MF alone; 2.MF/SVF; 3.MF/PRP; 4.Ringer’s lactate Control: non-irradiated	60Gy single exposure	Histology (H&E) Collagen density (Masson’s trichome and orcein staining) Vascularisation (number and diameter of dermal capillaries)	12wk post-graft	Greatest area of wound healing seen in MF+ PRP group No significant difference in epidermal thickness amongst the cell-based intervention groups. MF+SVF group showed the greatest increase in density and diameter of vessels	Combination therapies appear to be more effective than SVF or MF alone, likely through increased vascularisation and decreased subcutaneous sclerosis
**Borelli et al. [41]**	Collagenase digestion, filtration and centrifugation of human adipose tissue Whole fat and SVC isolated	Not applicable	Mice (n = 4) grafted post-radiation Interventions: 1.Whole fat/SVC; 2.Whole fat only; 3.PBS injection Control: sham (no injection)	30Gy fractionated into 6 doses of 5Gy (total = 12 days)	Limb extension (ruler) Tensile strength testing (microtester) Histology (H&E) Collagen density (Masson’s Trichrome, and Picrosirius Red) IF (CD31, CD26, Dlk-1, vimentin)	12wk post-graft	Greatest reduction in skin stiffness (less dense collagen) and greatest increase in elasticity in whole fat/SVC group Greatest increase in whole fat/SVC group (CD31) Significantly fewer CD26- and Dlk-1-positive fibroblasts in fat-grafted mice relative to control	The greatest functional and histological benefits were observed in mice receiving fat enriched with SVC
**Deleon et al. [42]**	Collagenase digestion, filtration and centrifugation of human adipose tissue ADSCs cultured (passage details not provided)	FACS (CD146, CD34)	Mice (n = 20) grafted post-radiation Interventions: 1.CD146+ ADSCs; 2. CD146- ADCs; 3.unfractionated ADSCs Control: fat only	30Gy fractionated into 6 doses of 5Gy given on alternate days (total = 12 days)	Fat graft volume (Micro CT) Mechanical strength testing (microtester) Histology (H&E) Collagen staining (Masson’s trichrome) IF (CD31, TGF-B, fibrillin, elastin, perilipin)	8wk post-graft	CD146+ADSC‐enriched fat graft group showed significantly more live adipocytes (perilipin) and vasculature (CD31), and showed the least collagen and TGF-β1 staining CD31+ enriched graft group showed the greatest improvement in skin elasticity (fibrillin and elastin)	The CD146+ ADSC subset comprises 1/3 of SVF, and may be responsible for the clinical effects observed with fat grafting in RIF
**Yao et al. [26]**	Collagenase digestion, filtration and centrifugation of rat adipose tissue ADSCs cultured to passage 3	Flow cytometry (CD10, CD34, CD45, CD73, CD90, and CD105)	Rats (n = 48) grafted post-radiation Intervention: ADSCs Control: PBS-injection	90Gy single exposure	Histology (H &E) Collagen staining (Masson’s trichrome and hydroxyproline) IHC (BCL2, CTSF, BAX) Western Blotting (CTSF, BAX, Bcl-xL, Caspase 9, GAPDH) Cell apoptosis (TUNEL assay)	12wk post- graft	Significantly lower lymphocyte count, collagen proportion and apoptotic cell count in ADSC-graft group compared to control Improved sebaceous gland and hair follicle regeneration in ADSC-graft group Expression of pro-apoptotic proteins significantly lower in ADSC-graft group relative to control	ADSCs inhibited lymphocyte migration, cell apoptosis and fibrosis formation In addition, gross skin histology was improved
**Khade-mi et al. [43]**	Collagenase digestion, filtration and centrifugation of rat adipose tissue ADSCs cultured to passage 3	No details provided	Rats (n = 32) grafted post-radiation Intervention: ADSCs Control: PBS injection	30Gy single exposure	Histology (H&E)	4wk post-graft	There were no significant differences in angiogenesis, fibrosis, collagen level, macrophage count, and epithelisation between the intervention and control groups Greater wound healing observed in intervention group	Lone ADSCs may not have an impact on alleviating RIF, and may require augmentation with PRP
**Riccobo-no et al. [44]**	Collagenase digestion, filtration and centrifugation of minipig adipose tissue Autologous ADSCs cultured (passage details not provided)	Flow cytometry (CD90, CD44, CD45 and CD31)	Minipigs (n = 6) grafted post-radiation Intervention: autologous ADSCs (intradermal and intramuscular injections) Control: PBS-injection	50.6 ± 4.1Gy single exposure	Western blotting (IL-6, IL‐1β, IL‐10) In-situ hybridization (TGF-β1, β-*actin*) IF (CD206/vWF, CD68 /CD206, CD80/CD206)	76 days post-radiation	TGF-β1 significantly increased in ADSC group, IL-10 detected only in ADSC group, and IL-6 and IL‐1β not detected in either Lower number of M1 macrophages (CD68, CD80) in ADSC group compared to control M2 macrophages (CD206) detected only in ADSC group	ADSCs may promote M2 macrophage polarisation, likely through IL-10 and TGF-β1 upregulation
**Riccobo-no et al. [45]**	Collagenase digestion, filtration and centrifugation of minipig adipose tissue Autologous ADSCs cultured	Flow cytometry (CD90, CD44, CD45, and CD31)	Minipigs (n = 6) Intervention: 1. Irradiated/autologous ADSCs (intradermal and intramuscular injections) Control: 1.Irradiated/PBS-injection; 2. Non-irradiated	50.6 ± 4.1Gy single exposure	Histology (H&E) IHC (α-SMA, collagen 1 and 3) Western blotting (IL-6, IL-1β, IL-10, SCA1, Pax7, Myf5, MyoD)	76 days post-radiation	IL-10 detected only in ADSC group, and IL-6 and IL‐1β not detected in either Two-fold greater intensity of quiescent satellite cell marker Pax7 in ADSC-treated animals in comparison to corresponding nonirradiated tissue Myf5 (overexpressed in activated satellite cells) was detected in all samples	ADSCs may promote M2 polarization through secretion of IL10 ADSCs may aid tissue repair through quiescent satellite cell recruitment and activation.
**Sun et al. [46]**	Collagenase digestion, filtration and centrifugation of rabbit adipose tissue ADSCs cultured to passage 3	Flow cytometry (CD34, CD31, and CD90)	Rabbits (n = 64) Intervention (intramuscular): Irradiated/ADSC-graft Controls (intramuscular): 1.Irradiated/PBS-injection; 2.Non-irradiated contralateral body sides of ADSC- and PBS-injected rabbits	80Gy single exposure	Collagen staining (Masson’s trichome) IHC (TGF-β1) Western blotting (TGF-β1) ADSC tracking (CM-Dil label)	26wk post-radiation	Significant decrease in collagen level and TGF-β1 expression in ADSC-treated group relative to control ADSCs were shown to disperse into surrounding muscular tissue	In irradiated muscle, ADSCs could alleviate RIF by suppressing TGF-β1 expression Further research in TGF-β1 knockout models is warranted
**Huang et al. [47]**	Collagenase digestion, filtration and centrifugation of rat adipose tissue ADSCs cultured to passages 4–6	FACS (CD29, CD21, CD34, CD45, CD90, CD73, CD31, vWF)	Rats (n = 16) grafted post-radiation Intervention: ADSCs Control: PBS-injection	50Gy single exposure	Histology (H&E) IF (vWF, CD31) ADSC tracking (CM-Dil label)	6wk post-radiation 2wk post-radiation	Capillary density (CD31, vWF) and thickness in the dermis were increased in the ADSC-treated group compared to control ADSCs co-localised with Ki67 (cell proliferation marker), CD31 and vWF At 2wk, ADSCs were clustered at the site of the injection but did not disperse into surrounding tissue	ADSCs could increase tissue survival and vasculature in RIF (either by fusing with or differentiating into vascular endothelial cells) Extended follow-up of CM-Dil labelled ADSCs required to track migration
**Chen et al. [48]**	Collagenase digestion, filtration and centrifugation of minipig adipose tissue Autologous ADSCs cultured to passage 3–5 PRF obtained from minipig blood sample	Flow cytometry (CD14, CD45, CD90)	Minipigs (n = 20) grafted post-radiation Interventions: 1.autologous ADSCs; 2.PRF; 3.ADSCs/PRF Control: PBS-injection	20Gy single exposure	Histology (H&E) IHC (Factor VIII) Cell apoptosis (TUNEL assay)	6mo post- final injection	The ADSCs/PRF combination group showed the highest adipocyte count and vascular density, as well as the lowest apoptotic density and fibrosis- and inflammation-associated characteristics. These differences relative to other groups were statistically significant	ADSC and PRF mixture achieved even better soft tissue regeneration than ADSCs or PRF alone Both may have similar mechanisms of action (neovascularisation, anti-apoptosis, structural remodelling)
**Evin et al. [49]**	Collagenase digestion, filtration and centrifugation of human adipose tissue ADSCs cultured (passage number unknown) PRP obtained from donor human patient	Flow cytometry (CD73, CD90, CD105, and CD45)	Mice (n = 24) each transplanted with an average of 14.5 human follicular units (FU). Grafted post-radiation to transplanted areas Interventions: 1.ADSCs/PRP; 2.ADSCs; 3.PRP Control: saline-injection	10Gy single exposure	Histology (H&E) IHC (Ki-67, Bcl-2, B-catenin) Western blotting (Bcl-2, Bax)	56 days post-radiation	Reduced atrophy, fibrosis, and skin keratinization in the ADSC-treated groups Highest proliferation (Ki-67), anti-apoptosis (Bcl-2), differentiation and signalling (beta-catenin) seen in FUs and skin of ADSCs/ PRP group, followed by ADSC-only and then PRP-only groups	ADSCs promote hair follicle regeneration, showing further potential for alleviating RIF (e.g., alopecia) and improving cosmetic outcomes PRP does not regenerate cells but may complement ADSC effects by providing a fibrin scaffold for repair
**Yu et al. [30]**	Collagenase digestion, filtration and centrifugation of human adipose tissue SVF obtained	Flow cytometry (CD29, CD11, CD73, CD45, CD105, CD90, CD34, HLA-DR)	Rats (n = unknown) grafted post-radiation Intervention: SVF cell suspension Control: PBS-injection	45Gy single exposure	Clinical score (pathological signs) Histology (H&E) IHC (IL-6 and TGF-β1)	72 days post-radiation	SVF-treated skin showed intact epidermal covering and less inflammatory infiltration compared to control Reduced IL-6 and increased TGF-β1 in SVF-treated skin tissues	SVF attenuated RIF through anti-inflammatory mechanisms
**Abbas et al. [50]**	Collagenase digestion, filtration and centrifugation of human adipose tissue Whole fat obtained	Not applicable	Mice (n = 7) grafted post-radiation Interventions: 1.Untreated fat graft 2.Vitamin E (VE)-treated graft 3.Pentoxifylline (PTX)-treated graft Control: nil grafting	30Gy fractionated into 6 doses of 5Gy given on alternate days (total = 12 days)	Histology (H&E) Collagen staining (Masson’s trichome) IHC (CD31, 8-isoprostane) Proteomic profile (Luminex)(including IFN-γ, IL-1, IL-2, CCL2/3/4/7/11, MIP-2, SRANKL, VEGF)	8wk post-graft	Significant decrease in dermal thickness and collagen density in VE- and PTX-treated groups compared to control VE-treated group showed greater CD31 staining, lower 8-isoprostane, and lower pro-inflammatory cytokine expression compared to all other interventions and control	Vitamin E contributes to graft survival and retention by reducing inflammation and oxidative injury, and assisting angiogenesis both within the graft and the overlying skin
**Kim et al. [51]**	Human ADSCs (details not provided) Salivary gland stem cells (SGSCs) and ADSCs seeded onto spheroids	Not provided	Mice (n = 30) Interventions (grafted into salivary glands 3wk post-radiation): 1.Matrigel only 2.Matrigel & SGSC/ADSC spheroids Controls: 1.Non-irradiated/PBS 2.Irradiated/PBS	15Gy single exposure	Salivary flow rate Body weight Gene expression (Microarray)(angiogenesis- related genes e.g., Vegfa) Histology (H&E, Masson’s trichrome) IHC (CD31, CD11b, CD68) Cell apoptosis (TUNEL assay)	12wk post-radiation	Higher salivary flow rate, lower fibrosis severity, lower apoptotic rate, and maintained cellular architecture and mucin secretion in irradiated/spheroid group compared to irradiated control Lower expression of CD68 (monocytes) and CD11b (leukocytes), and higher expression of CD31 and angiogenesis-related genes in spheroid group compared to other interventions	ADSC/SGSC-seeded spheroids show antifibrotic, anti-inflammatory, pro- angiogenic and anti-apoptotic effects Spheroid grafts may restore functionality in irradiated salivary glands
**Adem et al. [52]**	Collagenase digestion, filtration and centrifugation of human adipose tissue	Not applicable	Mice (n = 15) grafted 4wks post-radiation Interventions (grafted 4wk post-radiation): 1. Lipoaspirate 2.Reconstituted decellularized adipose matrices (DAM) Controls: 1.Non-irradiated/ non-grafted 2. Irradiated/ non-grafted	30Gy fractionated into 6 doses of 5Gy given on alternate days (total = 12 days)	Histology (H&E) Mechanical strength testing Collagen density (Masson’s Trichrome and Picrosirius Red) IF (CD31)	8wk post-graft	All intervention groups showed higher CD31 staining, and reduced skin stiffness (lower dermal thickness and collagen density) relative to irradiated controls Fat-grafted mice showed increased adipose integrity, higher CD31 staining, and lower fibrosis and collagen density relative to DAM-grafted mice	Fat-grafting had superior antifibrotic and angiogenic effects to DAM. However, DAM alone (biological scaffold enveloped in collagen, growth factors and cytokines) did show a positive effect
**Sowa et al. [53]**	Collagenase digestion, filtration and centrifugation of human adipose tissue Lipoaspirate and SVF obtained	Not applicable	Mice (n = 24) grafted 3wk post-radiation, followed by cutaneous punch wound 6mo post-graft Interventions: 1.Lipoaspirate 2.SVF 3.Micronized cellular adipose matrix (MCAM) Control 1.Sham (DMEM)	40Gy fractionated into four 10Gy doses weekly to delay and minimise acute radiation injury	Histology (H&E) Collagen density (Masson’s Trichrome) IHC (perilipin)	15 days post cutaneous wound	Intervention groups observed increased wound healing, however, no difference between groups Intervention groups showed reduced dermal hypertrophy, fat atrophy, and dermal fibrosis, with greatest effects in SVF and MCAM groups SVF and MCAM groups showed higher perilipin staining relative to lipoaspirate group	Adipose-derived grafts improve healing capacity of irradiated skin Adipose-derived grafts delivered shortly after radiation may offset chronic radiation fibrosis

Key.

AFT- autologous fat transfer.

α-SMA- alpha smooth muscle actin.

BAX- BCL2 Associated X Apoptosis Regulator.

BCL2- B-cell lymphoma 2.

Bcl-XL- B-cell lymphoma-extra large.

CCL- chemokine (C-C motif) ligand.

CTSF- cathepsin F.

Dlk-1- delta like non-anonical notch ligand 1.

FACS- Fluorescence-activated cell sorting.

GAPDH—glyceraldehyde-3-phosphate dehydrogenase.

GFP+/Luc+- Green fluorescent protein+/luciferase+.

IL-1β- i*nterleukin 1 beta*.

IL-6—interleukin 6.

IL-10—interleukin 10.

IOD- integrated optimal density.

Ki-67- Antigen KI-67.

MIP-2 –macrophage inhibitory protein 2.

Myf5- myogenic factor 5.

MyoD- myoblast determination protein 1.

Pax7- paired box 7.

PRF- platelet-rich fibrin.

PRP- platelet-rich plasma.

SCA1- stem cell antigen-1.

Shh- sonic hedgehog protein.

Smad3—SMAD family member 3.

SRANKL–soluble receptor activator of nuclear factor-kappaB ligand.

SVC- stromal vascular cell layer.

SVF- stromal vascular fraction.

TGF-β1—transforming growth factor beta *1*.

vWF—Von Willebrand factor.

### ADSC preparation and characterisation

Across all studies, ADSCs were prepared in a similar fashion. Adipose tissue was isolated from the target tissue and then digested with collagenases. After undergoing cycles of filtration and centrifugation, ADSCs were obtained and cultured through different passage numbers. In 18 studies, ADSCs were further characterised, and this was either achieved through flow cytometry, fluorescence-activated cell sorting (FACS) or differentiation assays to show ADSC cell surface markers or prove their differentiation potential.

### *In-vitro* study design

All nine *in vitro* experiments used a co-culture set-up where two different cell types were cultured together either directly in the same well or indirectly by using well inserts [23–31]. Four *in vitro* studies adopted ADSCs as their treatment. Only Xiao *et al*. utilised mature adipocytes. Shukla *et al*., Yao *et al*., Saijo *et al*., and Sörgel *et al*. used ADSC-cultured media (ADSC-CM). Yu *et al*. utilised SVF. A variety of different cell types were used in the co-culture experiments: human microvascular dermal endothelial cell (HDMEC) (n = 1), normal human dermal fibroblasts (NHDF)(n = 2), human foreskin fibroblasts (HFF)(n = 1), bone marrow derived stem cells (BMSC)(n = 1), human immortalized keratinocyte (HaCaT)(n = 3), normal human oral squamous epithelial cells (NOK)(n = 1), human dermal lymphatic endothelial cells (HDLEC)(n = 1), and human skin fibroblast cells (WS1)(n = 2). The radiation protocol generally involved a single exposure of 2–20 Grays (Gy) (median 6Gy), with some studies using varying radiation doses. The methodology of the different studies included proliferation assays (n = 4), enzyme-linked immunosorbent assays (ELISA) (n = 3), Western Blotting (n = 5), reverse transcriptase reverse transcription quantitative real-time polymerase chain reaction (RT-qPCR) (n = 2), and migration assays (n = 3). Other experiments included: tube formation assay, Luminex assay, Terminal deoxynucleotidyl transferase dUTP Nick-End Labelling (TUNEL) apoptotic cell assay and Tandem Mass Tag (TMT)- based proteomic quantification.

### *In-vivo* study design

In the twenty-five *in vivo* studies, different species were utilised: mice (n = 14), rats (n = 4), mini pigs (n = 6), rabbit (n = 1), and human tissue (n = 1) [26,27,30,32–54]. Fifteen studies utilised a single exposure of 10 to 90Gy (median: 50.6Gy) and seven studies a cumulative exposure of 30Gy fractionated as six 5Gy doses over 12 days. Experimental design usually involved irradiation of animals and a transitionary period for RIF to develop (median 25 days), followed by one/multiple AFT procedures involving ADSC, SVF, whole fat or microfat. Control groups were either given phosphate buffered saline (PBS) or ringer’s lactate solution. Two studies looked at the augmentation of lipotransfer using ADSC sub-types. Furthermore, two studies investigated supplementing platelet-rich plasma (PRP) to improve AFT outcomes.The average observational period across studies was 76 days. Outcomes recorded included clinical evaluation, histological assessment (n = 20), mechanical strength testing (n = 5), fat graft volume retention using micro-CT (n = 2), immunohistochemistry (IHC) (n = 15), immunofluorescence (IF) (n = 4), Picrosirius red collagen staining (n = 3) and Massons trichrome collagen staining (n = 8).

### Risk of bias assessment

For *in-vitro* studies (OHAT assessment tool), a low risk of bias was noted in the domains of experimental conditions, exposure characterisation, incomplete data, and reporting on all measured outcomes. Authors provided sufficient information on plate conditions, media and solvents, and they were similar across control and intervention groups. However, they score lower on domains two and five, only due to limited information on whether assessor blinding had taken place. Automated systems where the cells are transferred to assays without assessor handling would mitigate the need for blinding.

Across the *in-vivo* studies (SYRCLE assessment tool), there was a low risk of selection, attrition, and reporting biases highlighted. Authors ensured similar baseline animal characteristics, measured primary/secondary outcomes in all intervention and control groups, and included all animals in the final analyses. However, we noted that most studies had insufficient details to assess domains three to six e.g., allocation concealment and assessor blinding. As this issue was common, it was not grounds for exclusion, however, this is an area for future studies to improve on.

### *In-vitro* studies

#### Fibroblasts

As previously mentioned, three cell lines were used human skin fibroblasts (WS1), primary proliferating normal human dermal fibroblasts (NHDF), and human foreskin fibroblasts (HFF).

Neither co-culture of mature adipocytes with WS1 cells nor of ADSCs with NHDFs increased proliferation of the fibroblasts [25,28]. However, Shukla *et al*. observed that ADSC-CM treatment reduced NHDF hypermigration post-radiation by 17.5% in a scratch migration assay when compared to NHDFs without treatment [24]. On the contrary Xiao *et al*. found that co-culture with mature skin adipocytes increased the migration of WS1 cells [28].

Ejaz *et al*. demonstrated that co-culture of HFF and ADSCs led to a downregulation in TGF-β1 and Col-1 expression compared to irradiated monoculture (RT-qPCR) [27]. In a Luminex assay, HGF production was increased in irradiated co-culture compared to non-irradiated co-culture. Furthermore, addition of HGF protein in increasing concentrations to irradiated HFFs independently decreased TGF-B1 expression in a dose-dependent manner. Haubner *et al*. noted an increase in the secretion of matrix metalloproteinase (MMP) 1 and MMP2 but also tissue inhibitor of metalloproteinases (TIMP) 1 and TIMP2 when co-culturing ADSCs and NHDFs, relative to the non-irradiated control. Irradiated NHDFs in monoculture showed a significant reduction of MMP1 and an increase in MMP2 and TIMP2. Through TMT- based proteomic quantification, Yu *et al*. demonstrated significantly higher secretion of 239 proteins in WS1/ADSC co-culture relative to WS1 monoculture [30]. Of the upregulated proteins, 64% were extracellular proteins including MMP1 and collagen subtypes. Kyoto Encyclopaedia of Genes and Genomes (KEGG)-based pathway enrichment analysis revealed that the ECM receptor interaction and focal adhesion pathways were also significantly upregulated.

#### Endothelial cells

Saijo *et al*. discovered that co-culturing irradiated human dermal lymphatic endothelial cells (HDLECs) with ADSCs led to increased proliferation, compared to HDLEC monoculture [29]. They further noted that adding ADSC-CM resulted in smaller scratches (scratch migration assay) and approximately two-fold longer tubes (tube formation assay) [29]. Haubner *et al*. demonstrated that when co-culturing HDMECs with ADSCs, intercellular adhesion molecule 1 (ICAM-1), vascular cell adhesion protein 1 (VCAM-1), IL -6, and fibroblast growth factor 2 (FGF2) secretion were significantly reduced compared to HDMEC mono-culture [23].

#### Epithelial cells

Using a TUNEL apoptosis assay, Yao *et al*. demonstrated that the proportion of HaCaT and NOK undergoing apoptosis was lower in the ADSC-CM treated group than in the control group treated with normal medium [26]. Furthermore, the secretion of pro-apoptotic CTSF, BAX, Bid, and caspase 9 was downregulated in cells treated with ADSC culture supernatant (Western blotting). Xiao *et al*. showed that mature skin adipocytes in co-culture had no effect on keratinocyte proliferation but increased their migration [28]. On the other hand, Sörgel *et al*. reported that ADSC-CM had no impact on the viability and migration of keratinocytes [31].

#### BMSCs

Ejaz et al. noted that there was a significant downregulation in the expression of TGF-β1, collagen (COL)1, connective tissue growth factor (CTGF), tumour necrosis factor (TNF), nuclear factor kappa-light-chain-enhancer of activated B cells (NF-κB), and COL4 genes in ADSC/BMSC co-culture relative to BMSC monoculture [27].

### *In-vivo* studies

#### ECM remodelling

Sultan *et al*. noted that the fat-grafted animal group showed less Smad3 activity accompanied by a 42% lower scar index compared with the saline-grafted group eight weeks post-graft [33]. Similarly, Garza *et al*. discovered that irradiated mice scalp tissue displayed a significant decrease in collagen amount at eight weeks post-fat graft compared to irradiated tissue pre-fat graft [36]. However, Yu *et al*. showed that TGF-β1 levels (IHC) were increased and IL-6 levels decreased in SVF-grafted rats relative to PBS-grafted controls at 2 weeks post radiation [30].

Sun *et al*. showed that there was a decrease in collagen fibres (Masson’s trichrome) as well as lower amounts of TGF-β1 (IHC) in ADSC- treated rabbit muscle at 8 and 26 weeks compared to the PBS-treated control [46]. Yao *et al*. demonstrated that collagen fibre density (Masson’s trichrome) and hydroxyproline levels was significantly decreased in the ADSC-grafted rat group at 4- and 12-weeks post irradiation compared to the PBS control group [26]. Both Adem *et al*. and Sowa *et al*. showed reduced dermal thickness and collagen density in irradiated mice grafted with lipoaspirate, relative to controls [52,53].

On histological analysis, Khademi *et al*. found no difference in fibrosis and collagen level between ADSC- and PBS-injected rats [43]. Further, Ejaz *et al*. showed that collagen staining in ADSC-treated mice 30 days post-radiation was similar to non-irradiated control mouse skin [27]. By using GFP+ Luc+ ASDCs they additionally demonstrated that injected ADSCs were still present at the irradiated sites 77 days post-injection. However, Riccobono *et al*. noted increased staining of alpha smooth muscle actin (α-SMA) (IF) in ADSC-grafted muscle tissue when compared to the PBS-grafted group at day 76 [35].

#### Epithelial cells

Forcheron *et al*. showed that in ADSC-grafted animals, there was an increase in cytokeratin expression (IF) after irradiation [34]. This corresponded with increased proliferation of keratinocytes recorded at day 119, two weeks after the fourth ADSC graft (Ki-67 staining). Q-dot labelling revealed that ADSCs were not present in the epidermis.

#### Endothelial cells

Lindegren *et al*. demonstrated that AFT had the second greatest effect on the hypoxia gene pathway in irradiated breast tissue [39]. The authors noted decreases in CTFG, NDST1, FOSL2 and PPFIA4 mRNA gene expression alongside with increases in FAM162A, GPC3, INHA, GPC4 and FOX03 mRNA expression.

#### Immunomodulation

Kim *et al*. demonstrated lower expression of CD68 (monocyte lineage marker) and CD11b (leukocyte marker) in ADSC-seeded spheroid-grafted mice relative to irradiated controls [51]. Lindegren *et al*. noted that AFT decreased the macrophage density (CD86 IHC marker) in irradiated human breast tissue [39]. However, in contrast, Khademi *et al*. discovered on histological examination after 8 weeks that there was no difference in median macrophage number between ADSC- injected and PBS control-injected rats [43].

Riccobono *et al*. showed that IL-10 (Western Blotting) was present in irradiated muscle tissue in an ADSC-injected group, but not detected in the PBS-injected group at day 76 post irradiation [45]. Through IF, they also demonstrated that ADSC-treated muscle tissue stained strongly for M2 macrophage markers such as CD206, whilst weakly staining for M1 macrophage markers such as CD68 at day 76 post-irradiation [44].

Yao *et al*. found that upon histological examination, the number of lymphocytes in ADSC-treated rats at 4 and 12 weeks was lower than PBS-grafted controls [26]. Controversially, Forcheron *et al*. demonstrated earlier lymphocyte infiltration in ADSC-injected minipigs relative to the PBS-injected controls [34]. Using Fluorescent Quantum dot (Q-dot) labelled ADSCs, authors demonstrated amassing ADSCs at the subcutis/dermis junction post-grafting.

Lindegren *et al*. demonstrated that AFT greatly suppressed the pro-inflammatory interferon-γ gene pathway in irradiated breasts [39].

#### BMSCs

Ejaz *et al*. generated GFP^+^ Luc^+^ bone marrow chimeric mice through total body irradiation and subsequent bone marrow transplantation [27]. Four weeks later, one leg was irradiated and injected with ADSCs, whilst the contralateral leg served as a control. GFP^+^ Luc^+^ bone marrow-derived stem cells were detected at the injection site 21 days post-irradiation but not detected in the non-irradiated contralateral leg. FACS analysis of irradiated tissue demonstrated an increase in cells expressing CD34 and Sca1 (haematopoietic), CD45 (lymphocytes), and CD31 (VEC).

#### Vascularisation

Garza *et al*. demonstrated increased CD31 staining in irradiated mice scalp tissue at two- and eight-weeks post-fat grafting compared with irradiated tissue prior to fat grafting [36]. Adem *et al*. showed higher CD31 staining (IHC) in fat-grafted mice compared to irradiated controls [52]. Kim *et al*. noted higher CD31 staining (IHC) and pro-angiogenic gene expression in ADSC-seeded spheroid-grafted mice compared to controls [51]. Huang *et al*. also noted increased CD31 staining (IHC) and vascular density in the ASDC-treated rat group compared with the control group three weeks after fat grafting [47]. ADSCs utilised in the experiment were labelled with CM-Dil to track movement two weeks after injections. Authors noted that a proportion of labelled ADSCs co-localized with the vascular markers von Willebrand factor (vWF) and CD31, which were found in dendritic vascular structures.

On the contrary, Forcheron *et al*. demonstrated that after wound healing was complete in ADSC-grafted mini pigs, there was no evidence of increased vascular proliferation [34]. Khademi *et al*. showed that there was no significant difference in angiogenesis between rats injected with PBS and ADSCs at four weeks post-grafting [43]. Sultan *et al*. discovered that there was a significant decrease in CD31 staining (IHC) in fat-grafted mice compared to the saline-grafted mice at eight weeks post-grafting, while no significant difference was observed at four weeks post-grafting [33]. However, both sham and fat-grafted animals demonstrated greater vascular density than non-irradiated controls.

#### Augmentation of AFT

Luan *et al*. enriched their fat grafts with ADSCs, known as cell-assisted lipotransfer (CAL). Results included reduced collagen staining (Masson’s trichrome), increased CD31 staining (IHC), and decreased skin stiffness at eight weeks post-grafting in mice compared to fat alone [38].

As shown by Abbas *et al*., Vitamin E-treated fat grafts resulted in greater CD31 staining, lower 8-isoprostane (oxidative injury marker), and lower pro-inflammatory cytokine expression compared to fat grafts without Vitamin E treatment [50]. Pentoxifylline treatment also reduced dermal thickness and collagen density.

Bertrand *et al*. demonstrated that, in mice injected with microfat and SVF, there was greater increase in diameter and density of vessel at 12 weeks post-graft than microfat alone [40]. Furthermore, on average a greater wound area underwent successful healing in the microfat/SVF-injected subgroup compared to the microfat-injected subgroup. In the study by Sowa *et al*., the SVF-injected subgroup showed reduced periphilin staining relative to the fat-only subgroup [53].

Borrelli *et al*. demonstrated that grafting with fat enriched with stromal vascular cells (SVC) resulted in less dense collagen staining than mice receiving saline, sham injection, or fat-alone treatment at 12 weeks post-graft (Masson’s trichrome) [41]. There was also increased CD31 staining (IHC) in fat-grafted mice relative to sham or saline injection, However, there was no significant difference between fat-only and fat/SVC subgroups. Furthermore, immunostaining showed decreased CD26+ and protein delta homolog 1 (Dlk+) fibroblast subpopulations in fat grafted mice relative to sham injection. However, there was no difference noted in CD26+ and Dlk staining between fat/SVC and fat-only subgroups.

Evin *et al*. and Bertrand *et al*. studied the effects of PRP augmentation [40,49]. The former demonstrated the highest activation of proliferation (Ki-67), anti-apoptosis (Bcl-2), differentiation and signalling (β-catenin) within hair follicles by the ADSCs+ PRP subgroup, followed by ADSCs only. The latter reported the greatest area of wound healing in their microfat/PRP subgroup compared to both microfat/SVF and microfat-injected subgroups.

Chen *et al*. investigated the effects of platelet-rich fibrin (PRF) augmentation on irradiated minipig parotid glands, compared to ADSC-only, PRF-only and sham control groups [48]. Six months post-injection, defects in the combined group were the smallest and shallowest. This group also showed the highest rate of neoangiogenesis (Factor 8) and anti-apoptosis (TUNEL assay).

Kim *et al*., Sowa *et al*., and Adem *et al*. studied the impact of 3-D biological structures on graft outcomes, with regards to ADSC-seeded spheroids, micronized cellular adipose matrices (MCAM), and decellularized adipose matrices (DAM) respectively [51–53]. In irradiated murine salivary glands, spheroid treatment appeared to restore both cellular architecture and functionality (mucin secretion). MCAM-grafted tissue displayed reduced dermal hypertrophy, fat atrophy and dermal fibrosis relative to lipoaspirate and controls. DAM-grafted tissue had increasedCD31 staining and lower collagen staining compared to irradiated control, however, fat grafting was superior.

#### ADSC subpopulations

With use of FACS, some studies sought to further characterise ADSC by their sub-populations and test their efficacy in AFTs individually. For example, in irradiated mice Borelli *et al*. compared fat graft injections (200 μL) enriched with either CD74+, CD74−, unsorted ADSCs (CD34+ only) and a lipoaspirate-only control [32]. The authors discovered that CD74+ enrichment led to significantly decreased collagen (Masson’s trichrome) and Versican staining (IF) as well as skin stiffness at eight weeks compared to other sub-groups. Through FACS, CD74+ enrichment also significantly decreased the proportion of fibrotic papillary and reticular fibroblasts, and significantly increased the proportions of anti-fibrotic zigzag and lipofibroblasts [55]. Finally, fat grafts enriched with CD74+ led to reduced inflammation, increased integrity and less fibrosis compared to all other sub-groups on histological analysis.

Deleon *et al*. also characterised and tested ADSC sub-populations with enrichment for CD146 in fat graft (200 μl) injections [42]. Grafts enriched with CD146+ were noted to have significantly increased staining for fibrillin, perilipin and CD31. In addition, this sub-group showed the greatest decrease in collagen (Masson’s trichrome), TGF-β1 and elastin staining compared to other sub-groups at eight weeks post-fat grafting.

Riccobono *et al*. compared autologous ADSC grafts to allogenic ADSC grafts in a mini-pig cutaneous radiation model. Using a clinical score, autologous ADSCs were noted to have improved radiation wound healing at the final study endpoint relative to the PBS control group, however, allogenic ADSCs had not [35].

## Discussion

**Fig 2:**
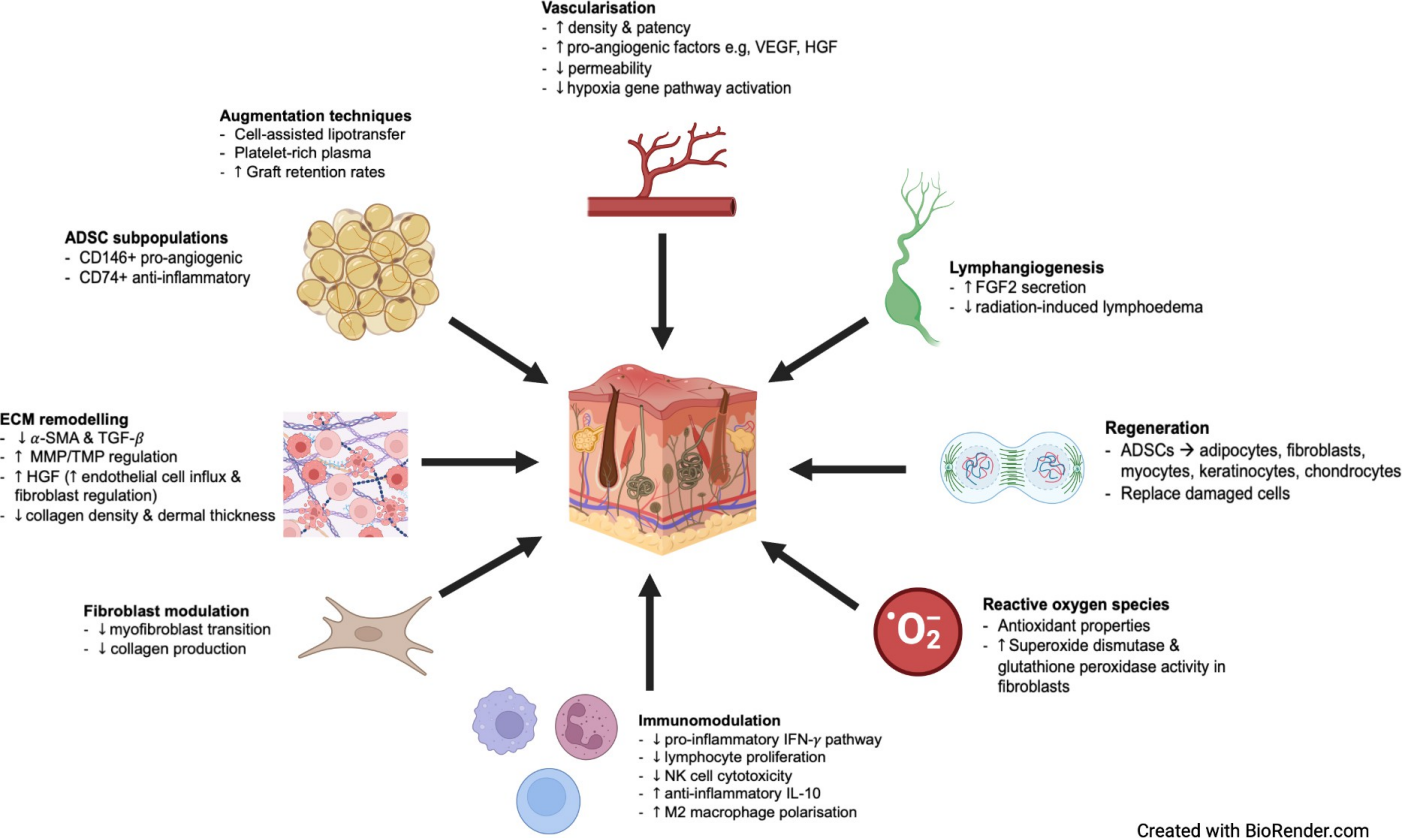
Diagrammatic summary of AFT mechanisms of action. AFT treats RIF through various mechanisms of action, including improved vascularisation, lymphangiogenesis, cell regeneration, immunomodulation, fibroblast modulation and ECM remodelling. Certain ADSC subpopulations have been shown to possess unique beneficial characteristics. Augmentation techniques such as cell-assisted lipotransfer and platelet-rich plasma have also shown promise.

This systematic review has summarised *in-vitro* and *in-vivo* findings on the effect of AFTs, but also graft-inherent cell types such as ADSCs on RIF of the skin (Fig 2). *In-vitro* research has mainly focused on the impact of ADSCs with very limited research on other cell types within the fat graft, such as endothelial cells, smooth muscle cells, preadipocytes and fibroblasts [56]. For *in-vivo* research, whole fat, micro fat, SVF as well as ADSCs alone were investigated for efficacy. Modifications of the fat grafts such as CAL with isolated ADSCs led to improved outcomes [32,38,40]. An interest in ADSC sub-populations led to findings of CD146+ and CD74+ ADSCs possibly being more effective [41,42].

In the following, we will discuss different possible effects of AFTs and its inherent cell types on RIF of the skin. These can be roughly categorized into effects on vascularisation, immunomodulation, and the extracellular matrix.

### Vascularisation

Radiotherapy is known to induce a local inflammatory microenvinroment with increased vascular endothelial dysfunction and permeability. A microscopic hallmark of RIF is microvascular obliteration–a consequence of induced local inflammation and excessive collagen deposition. The disruption and loss of micro-vessels in combination with an increased demand for oxygen, due to an influx of inflammatory and mesenchymal cells to the site of injury, leads to the accumulation of reactive oxygen species (ROS). While ROS are necessary due to their antimicrobial activity in normal wound healing, they cause damage to the tissue in the dysregulated fibrotic environment. Furthermore, ROS strongly activate the TGF-β/SMAD axis and lead to additional downstream collagen accumulation [57]. Hypoxia, caused by post-radiation vasculopathy, is considered a major stimulus of the fibrotic response seen in RIF [58]. Hence, it would seem beneficial for AFTs to enhance tissue vascularisation to counteract this phenomenon.

However, it may not be a question of vascular density but rather the quality and functionality of the present vasculature, indicated for example by their patency [59]. Sultan *et al*. found increased vascular density four weeks after irradiation injury compared to non-irradiated controls, indicated by increased staining for CD31 and vWF both in sham and fat-grafted mice [60]. In addition, levels of pro-angiogenic molecules such as vascular-endothelial growth factor (VEGF) and stromal cell-derived factor-1 (SDF-1) were higher in the fat-grafted group [60]. After eight weeks, however, less CD31 and vWF were measured in the fat-grafted group, while presenting with a 42% lower scar index compared to the sham-grafted group. This possibly indicates that AFT promotes more proficient vascularisation, leading to closer resemblance to normal skin in the pre-irradiation state. Similarly in other scarring and fibrotic skin conditions, AFT normalized abnormal microvasculature architecture [61,62]. In addition, Lindegren *et al*. studied genetic pathways in irradiated human breast samples, finding that the hypoxia gene pathway was severely aberrant after radiation and AFT restored this back to normal [39].

ADSCs alone showed ambiguous effects on vascularisation *in vivo*. One study noted them to increase vascular density three weeks post-grafting, while another study claimed there was no difference between PBS- and ADSC-injected rats after four weeks [43,47]. Huang *et al*. showed that ADSCs co-localized with vWF and CD31 markers at day 14 post-fat graft [47]. Unrelated to RIF of the skin, Nie *et al*. demonstrated similar findings using an excisional wound healing model [63]. In this study, ADSCs activated wound healing processes and angiogenesis, as well as being present in newly formed capillaries. It remained unclear whether the ADSCs directly differentiated into VEC, increased VEC proliferation, or supported VEC function by fusing with them. Although the potential of ADSCs to differentiate into VEC *in vivo* has been demonstrated repeatedly, it remains unclear if this mechanism attenuates RIF of the skin by promoting or regulating angiogenesis [63,64].

Another potential way ADSCs may promote angiogenesis is through the secretion of growth factors at the injection site. Previous research has proven the ability of ADSCs to secrete proangiogenic factors including different VEGFs as well as basic fibroblast growth factor (FGF2) and hepatocyte growth factor (HGF), which have also been found to promote angiogenesis [23,63,65,66]. HGF may play an important role in the angiogenic potential of ADSCs. Cai *et al*. achieved HGF-knockdown in ADSCs and observed a significantly reduced capacity to promote endothelial cell maturation, migration, and angiogenesis in a mouse hindlimb ischemia model [67]. Yet, no *in vivo* study has examined the expression of HGF and VEGF in skin harvested from fat-grafted animals for RIF. This warrants further investigation. Haubner *et al*. also found that FGF2 levels were increased in ADSC and HDMEC co-culture experiments, however, the relative increase was lower in the co-culture as compared to ADSCs alone [23]. Whilst this does suggest a role for FGF2, overexpression might be counterproductive with ADSCs potentially regulating ideal levels. Similarly, FGF2 is believed to have positive effects on wound healing by accelerating proliferation and granulation, while overexpression may lead to uncontrolled formation of granulation tissue [68].

Radiotherapy is known to induce a local inflammatory microenvinroment with increased vascular endothelial dysfunction and permeability. Coupled with the secretion of pro-inflammatory chemotactic cyotkines such as IL-6, leukocytes adhere to and transmigrate across the vascular endothelium into surrounding cells with more ease [69]. In particular, monocyte chemoattractant protein-1 (MCP-1) and IL-8 can trigger firm adhesion of monocytes to the vascular endothelium. Haubner *et al*. also noted that radiation-induced HDMEC dysfunction involved the upregulation of pro-atherogenic adhesion molecules ICAM-1 and VCAM-1 [70]. This upregulation was attenuated by ADSC co-culture, as well as IL-6 expression. ADSCs could help ameliorate VEC dysfunction in this way.

### Lymphangiogenesis

Radiotherapy is also an independent risk factor for developing lymphoedema, and ADSCs may also play a significant role in lymphangiogenesis post-irradiation [71]. Saijo *et al*. demonstrated that, when co-cultured with ADSCs and ADSC-CM, irradiated HDLECs efficiently proliferated, migrated, and formed tubes [29]. Outside of our study sample, Ahmadzadeh *et al*. showed a similar effect of ADSCs on non-irradiated HDLECs. They demonstrated more effective proliferation, migration and formation of tube-like structures than after stimulation with either VEGF-C, HGF or FGF alone–suggesting the combination of factors in the ADSC-CM to be most effective [72]. Saijo *et al*. theorised a paracrine mechanism of action through upregulation of FGF2, which is both a pro-lymphangiogenic and pro-angiogenic factor. FGF2-knockdown in these HDLECs significantly decreased its lymphangiogenic capabilities.

### Reactive oxygen species

Outside of our study sample, Kim et al. measured the anti-oxidative properties of ADSC-CM and found it be equally potent as 100 μM ascorbic acid [73]. They treated human fibroblasts with *tert­*-butyl hydroperoxide (mimicking the oxidising effects of radiotherapy) and incubated them with ADSC-CM, resulting in improved survival and increased SOD and glutathione peroxidase (GPx) activity in fibroblasts. Proteomic analysis of the cultured media further showed different anti-oxidative compounds including superoxide dismutase (SOD), insulin-like growth factor (IGF), hepatocyte growth factor (HGF), platelet-derived growth factor (PDGF), keratinocyte growth factor (KGF), and FGF among others.

### Immunomodulation

Cytokines, chemokines, and damage-associated molecular patterns (DAMPs) are produced by epithelial and endothelial cells damaged by radiation. This causes an influx of inflammatory cells, predominately macrophages and neutrophils, which are supposed to eliminate dead cells, debris, and invading pathogens [2,74]. Repetitive trauma or radiation and a disproportionate immune response with prolonged, unresolved inflammation ultimately lead to excessive amounts of ECM deposition [2]. Macrophages are usually found near fibroblasts and repeatedly described as master regulators of fibrosis [75,76]. Depending on their phenotype, they have diverse functions ranging from coordinating wound healing by promoting angiogenesis, tissue remodelling, and resolving inflammation (M2 type), to recruiting immune cells, causing inflammation, and tissue destruction (M1 type) [77]. However, while early fibrosis is characterised by high numbers of inflammatory cells and activated, large fibroblasts with less compact collagen fibres, later stages of fibrosis tend to show compact and organized collagen fibres and often less inflammation with less inflammatory immune cells [78]. Hence, possible immunomodulatory effects of AFT may also depend on what state of RIF is present–early vs late.

The SVF as part of a lipotransfer consists of a variety of cell types including macrophages, endothelial cells, ADSCs, and so on. However, lymphocytic immune cells account for the largest group of cells in the SVF [79,80]. This results in a variety of possible mechanisms by which AFTs can restore tissue homeostasis in RIF. The immunomodulatory effects of AFTs and its inherent cell types have been investigated in several studies. In the context of radiation injury, Lindegren *et al*. explored the expression of a multitude of gene pathways in irradiated human breast samples, finding that the interferon-gamma (IFN-γ) gene pathway was severely dysregulated post-radiation and subsequently downregulated the most by AFT [39]. IFN-γ is a pro-inflammatory cytokine secreted by T helper 1 (Th1) cells and heavily involved in the development of RIF [81–83].

ADSCs have demonstrated great immunomodulatory capacity. They have been found to suppress lymphocyte proliferation, suppress natural killer (NK) cell cytotoxicity, inhibit differentiation of monocyte-derived immature dendritic cells, and promote macrophage polarization towards an anti-inflammatory M2 phenotype [26,84]. In RIF, some of these effects have been reproduced. In irradiated minipig muscle, Riccobono *et al*. displayed that ADSC injection led to a greater proportion of M2 to M1 macrophages relative to irradiated PBS-injected control, as well as higher amounts of IL-10 and TGF-β [44]. Both IL-10 and TFG-β have been linked to M2 polarization, however, TGF-β is also one of the main drivers of tissue fibrosis [85]. Other study settings have proposed IL-6 and colony-stimulating factor 1 (CSF1) as possible mediators of ADSC-induced M2 polarization [86,87]. These M2 macrophages then help drive repair of radiated tissues. If large numbers of M1 macrophages were present, they would likely to shift the skin from a state of repair to continued inflammation, myofibroblast survival, and fibrosis [44,88].

M2 macrophages can be split further into sub-types (M2a, M2b, M2c and M2d) with varying roles [85]. Further research is required into these specific subtypes and how ADSCs can modulate their function, since literature to date has only focused on M2 macrophages as a collective whole. Furthermore, expression of tumour necrosis factor-inducible gene 6 protein (TSG-6) and other anti-inflammatory chemokines should be studied in greater detail. In microglia, Hu *et al*. demonstrated that ADSC-secreted micro-RNAs upregulated TSG-6 expression, which then inhibited immune-mediated inflammation and encouraged tissue repair [89]. Whilst this immunomodulation shows promise in future treatment of inflammatory central nervous disorders such as multiple sclerosis, it is not known whether this applies to RIF.

### Extracellular matrix remodelling

Excessive extracellular matrix (ECM) deposition–especially collagen–is the hallmark feature of fibrosis. Remodelling of the ECM after injury is essential for tissue homeostasis and in the case of RIF is strongly dysregulated with fibroblasts producing vast amounts of ECM. AFTs could possibly remodel the ECM directly through ECM synthesis, degradation, and modification or indirectly by changing the behaviour of tissue resident cells which produce ECM e.g., fibroblasts.

AFTs have been shown to reduce, remodel, and realign collagen fibres, as well as reduce α-SMA, dermal thickness and scar size [18]. For RIF of the skin specifically, this review provides further evidence that collagen density and dermal thickness significantly decrease after fat grafting (20, 23, 27) as well as ADSC injections alone (32). ADSCs have shown to prevent fibroblast to myofibroblast transition, cause myofibroblast apoptosis as well as generally decrease the production of collagen from fibroblasts in research of fibrotic skin conditions and scarring [18,19,90]. Further, ADSCs in co-culture with irradiated fibroblasts led to a decrease in α-SMA, collagen, and TGF-β production [23,27].

Secreted HGF, as well as FGF2, have been suggested as possible ways by which ADSCs can modulate fibroblast activity and remodel the ECM. They are believed to be part of an antifibrotic FGF2-JNK-HGF pathway [91]. FGF2 supposedly causes myofibroblast apoptosis and inhibits the TGF-β1/Smad–pathway with subsequent reduction of α-SMA expression and collagen production. It is also believed to increase MMP-1 and decrease TIMP-1 [92–94]. FGF2 administration to hypertrophic scars in animal models reduced epidermal thickness and collagen levels [95]. HGF was present in ADSC monoculture alone and co-culture of ADSCs with irradiated fibroblasts increased HGF levels. When recombinant HGF was added to an irradiated fibroblast monoculture, a dose-dependent reduction in TGF-β levels was also recorded [27]. According to Ejaz *et al*., ADSC-secreted HGF may also stimulate the influx of endothelial, bone marrow stem cells and other cell types that collectively remodel the extracellular matrix [27]. The authors showed an influx of GFP^+^ Luc^+^ labelled BMSC in AFT-treated mice, but none were identified in saline-grafted mice. HGF has been shown to recruit BMSCs via interactions with their c-met receptor [96]. After they are recruited, ADSCs may affect the expression profiles of these BMSCs, as Ejaz *et al*. noted significant downregulation in the expression of pro-fibrotic proteins (TGF-β, COL1, CTGF) in ADSC/BMSC co-culture.

Irradiation leads to a dysregulation of MMPs and TIMPs–a further driver of RIF [97,98]. In chronic RIF of the skin, it would seem beneficial if AFTs or ADSCs would increase the amount of active MMPs while decreasing TIMPs. This would enable tissue remodelling with a decrease in ECM. Indeed, a liquid chromatography (LC-MS) study of the ADSC secretome revealed the highest enrichment score and protein counts for ECM proteins, cell adhesion proteins as well as MMPs and TIMPs [95]. However, the functions of TIMPs and MMPs go beyond tissue remodelling and their regulation is complex [99]. They can promote inflammation as well as decrease inflammation. Further, MMPs are tissue specific, regulated by immune and stromal cells, and their functions depend on disease and tissue context [100]. For example, some MMPs–such as MMP8 –are profibrotic while others are believed to be antifibrotic [99]. Further, ECM degradation by MMPs also leads to the release of dormant TGF-β. Excessive MMP activity mat even facilitate the development of chronic wounds, by breaking down newly formed ECM of granulation tissue too quickly, before healing can begin [101]. Hence, it is not as simple as upregulating MMPs and downregulating TIMPs to treat fibrosis. TGF-β decreases the activity of MMPs, specifically MMP-2, MMP-9, and MMP-1, within fibroblasts [102,103]. ADSCs counteracted this effect with increased MMP1 and MMP2 levels, but also increased TIMP1 and TIMP2 levels detected in co-cultures with irradiated fibroblasts [25].

The dynamics of ECM remodelling in fibrotic skin conditions are complex and no simple answer exists on how AFTs or ADSCs may reinstate tissue homeostasis. However, we assume RIF is counteracted by reinstating the physiological balance between ECM synthesis and degradation. This is achieved directly by downregulating profibrotic MMPs and secreting anti-fibrotic MMPs, as well as indirectly by inhibiting excessive ECM production from fibroblasts. This is by preventing their activation and transition into myofibroblasts.

### Regenerative ability

The variety of cell types such as endothelial cells or immune in AFTs could possibly replace damaged cells in RIF of the skin. Here, emphasis has been placed mainly on ADSCs. As a form of mesenchymal stem cell (MSCs) they are capable of self-renewal, show long-term viability, and multilineage potential. They can differentiate into various cell types of the mesenchymal lineage including adipocytes, fibroblasts, myocytes, keratinocytes, chondrocytes, osteoblasts but also of other lineages such as neural cells, endothelial cells, and hepatocytes [104,105]. An *in vivo* study by Haubner *et al*. showed that when ADSCs were cultured in HDMEC medium, ADSC count decreased progressively during the follow-up period. One suggested theory was the ADSCs were actively differentiating into endothelial cells [23]. Hence, ADSCs could possibly directly replace cell types damaged by radiation. It remains unclear if this mechanism is part of the clinical effect observed after AFTs.

### Anti-apoptosis

Radiation-induced cell death of epithelial and endothelial cells is a key driver of RIF of the skin. This leads to the production of cytokines, chemokines, and DAMPs, and subsequent inflammation and influx of immune cells; all of which impair tissue function [106]. AFT may decrease cell death and henceforth prevent further disease progression. When added to irradiated fibroblasts, SVF upregulated DMTF1, MFAP5, and P116, all of which regulate key cellular apoptotic pathways [30]. Similarly, ADSCs led to downregulation of pro-apoptotic proteins (CTSF, Bid, BAX) and fewer cells undergoing apoptosis as a result in co-culture with human immortalized keratinocytes (HaCat) and human oral squamous epithelial cells (Nok) (14). Evin *et al*. showed increased anti-apoptotic Bcl-2 expression by hair follicles was in their ADSC/PRP and ADSC groups, relative to the control [49]. Rehman *et al*. also reported direct secretion of anti-apoptotic factors by ADSCs [66]. Huang *et al*. showed that injected ADSCs co-localized with the cell proliferation marker Ki67 *in vivo*, providing further evidence that ADSCs may promote cell survival and preserve tissue function and integrity [47].

### Augmentation of fat grafts

A prominent limitation of fat grafting in RIF is its retention in hostile, irradiated tissue. In studies of autologous fat grafts in non-irradiated sites, retention rates already vary between 40–60% [107]. On the other hand, irradiated tissue is a fibrotic, hypovascular and inflammatory environment. Grafted fat is therefore susceptible to central necrosis and subsequent inflammation [15]. This can lead to cyst formation, further fibrosis and local infection [108]. Augmentation techniques may help overcome these limitations.

CAL is the enrichment of fat grafts with cells from SVF or with culture-expanded ADSCs. This technique has already shown efficacy in human trials for autologous fat grafts and cosmetic breast augmentation [109,110]. CAL resulted in greater fat volume retention, reduced post-operative atrophy and better cosmetic outcomes. Recently, Luan *et al*. has also demonstrated its efficacy in RIF treatment, with regards to reduced collagen deposition and increased angiogenesis (CD31) [38].

SVF consists of several cell types, including pericytes, endothelial, haematopoietic, stromal and stem cells. ADSCs only make up less than 5% of SVF. As Yu *et al*. demonstrated, all these cell types can affect the extracellular matrix through various pathways. Upregulated proteins included microfibrillar-associated proteins (MFAPs) and MMP-1 which complement the regenerative effects of ADSCs [30]. Practical advantages of using SVF over ADSCs alone stems from its simpler isolation process, meaning higher yields can be produced. In addition, no ex-vivo amplification is required which thereby reduces the risk of cell contamination and potential neoplasia [111].

PRP is a concentrate of platelets, obtained after centrifugation of whole blood. It is abundant in pro-regenerative growth factors including PDGF, TGFB, VEGF and epidermal growth factor [49]. Augmentation with PRP is an exciting area of research because its fibrin provides a three-dimensional structure which helps to retain both ADSCs and their secreted growth factors. This allows for appropriate cell migration and enhanced paracrine effects of these growth factors [112]. In particular, the work by Evin *et al*. on the effects of ADSC/PRP on irradiated hair follicles is promising for the treatment of radiation-induced alopecia in head and neck cancer. The potential benefits of PRF goes even further. This is a second-generation platelet concentrate which is easier to prepare, less expensive and carries a lower risk of immunological rejection compared to PRP [113].

Following from above, several bioactive structural supports in injectable form are currently under investigation, in attempts to better simulate ECM properties. In particular, in vitro research with conventional monolayer cell cultures is significantly limited, as it cannot recreate the mechanical and biochemical communication networks which aid ADSC proliferation and differentiation. Decellularized adipose matrices (DAMs) are acellular, albeit natural 3-D structures that have shown promise even if they do not resemble the donor site’s anatomy [114]. In our included study by Adem *et al*., demonstrated retention of anti-fibrotic cytokines FGF-2 and MIF, and pro-angiogenic cytokines such as PDGF-AA [52,115]. However, DAMs were inferior to fat grafting alone. This highlights the importance of ADSCs in facilitating de-novo adipogenesis and tissue regeneration. DAMs enriched with further growth factors and ADSCs to levels higher than native grafts may be an avenue to explore. For example, micronized cellular adipose matrices (MCAMs) contain a high proportion of ADSCs, minimal adipocytes and some capillaries [116]. Sowa *et al*. demonstrated MCAMs had similar anti-fibrotic effects to SVF and superior adipogenic capability to fat grafting alone [53]. In addition, the preparation of MCAMs is less intensive, quicker, and not subject to the same regulations as SVF. Finally, Kim *et al*. proved the efficacy of cultured, ADSC-seeded spheroids in restoring functionality of irradiated murine salivary glands [51]. Prior to transplant, these cellular microenvironments and its biochemical pathways can be sequentially engineered. For example, the authors treated the spheroids initially with a Wnt/β-catenin pathway activator followed by FGF7 at intervals, to stimulate ADSC differentiation into salivary gland stem cells and paracrine factor secretion respectively.

Additional supplementation of these structures with biocompatible materials and compounds may prove beneficial. Research has shown how ADSCs embedded fibrin glue can enhance wound healing through stimulating angiogenesis, fibroblast migration and ECM organisation [117]. Abbas *et al*. have highlighted the potent anti-inflammatory and anti-oxidative effects of Vitamin E [50]. Although it has no pro-angiogenic properties itself, Vitamin E likely enhanced ADSC-induced angiogenesis through the above effects on the local microenvironment. In addition, its fat solubility is inherently advantageous in lipotransfer.

### ADSC subpopulations

Although often considered as a single entity, ADSCs are a heterogeneous mix of subpopulations with different cellular functions and secretomes. Previous research has identified angiogenic (CD248+), adipogenic (BMPR-1A+), osteogenic (CD105-/‘endoglin’) ADSCs [107]. The CD90+/CD73+/CD105+/CD45-/CD31-/ CD146+(−) sub-types are also known to secrete a large variety of ECM proteins [95].

In our study sample, Borrelli *et al*. identified promising antifibrotic and anti-inflammatory potential of CD74+ ADSCs in RIF [32]. CD74 activates the anti-inflammatory AMPK signalling pathway through the macrophage inhibitory factor (MIF) receptor [118]. The antifibrotic effects were theorised to be a result of increased fibrillin expression. Fibrillin-1 proteins form the structure of connective tissue microfibrils and are known to regulate TGF-β levels in the ECM [119]. Mutations in these proteins, pathognomonic to Marfan’s syndrome, results in elevated TGF-β and increased susceptibility to lung and kidney fibrosis in particular [120,121]. However, its exact role in RIF of the skin is unclear.

Deleon *et al*. noted increased expression of CD31 (angiogenesis), perilipin (adipogenesis) and fibrillin by CD146+ ADSCs [42]. The authors theorised that increased fibrillin expression may have enhanced elasticity of irradiated tissue overlying their CD146+ ASC-enriched fat grafts. Perilipin 1-deficient adipocytes have been previously shown to promote inflammation by stimulating monocyte migration and IL-6, iNOS, and IL-1*β* expression by macrophages [122]. Increased perilipin expression may counteract these inflammatory changes. Continued research into CD146+ ADSCs has shown further proangiogenic potential, through upregulation of VEGF and ANGPTI [123]. The latter stabilises VEGF-induced vascular endothelial cell proliferation and prevents blood vessel leakage [124].

Further proteomic studies are warranted to investigate individual ADSC subtypes (categorised by cell surface markers) and how they can be applied to treatment of RIF of the skin. These purified cells may improve lipotransfer outcomes whilst reducing potential side effects.

## Future advancements

Autologous fat grafting shows great potential for treatment of RIF, where it has been proven as a safe and effective treatment [17]. However, although many mechanisms of action of AFTs and especially ADSCs in attenuating RIF have been proposed, a comprehensive explanation of the underlying antifibrotic mechanism is still missing. The pathophysiology of RIF of the skin is complex and whether findings from other fibrotic skin conditions such as scleroderma can be adopted, remains to be proven.

Strikingly, most research has focused on the effects of ADSCs on fibrosis, while they only account for less than 5% of cells in the SVF [125]. They show undoubtable potential for regenerative medicine and this review has once again demonstrated their ability to seemingly attenuate hallmark features of fibrosis both *in vitro* as well as *in vivo*. However, future research should adopt a more wholesome approach to investigating how other cell types in the transplanted fat grafts could attenuate fibrosis. This includes macrophages, T cells, but also endothelial cells or preadipocytes. It also remains to be investigated whether ADSCs are a more or less effective treatment option than whole fat grafts. Furthermore, rising importance is being placed on identifying subsets of ADSCs, because they have been shown to be a heterogenous mixture of cells with different subpopulations. Identifying more effective subsets could further improve treatment efficacy and give insight into fibrotic and inflammatory pathways.

Large scale ex-vivo expansion of ADSCs and/or other stem cell types suitable for human use is a significant barrier for transition into mainstream clinical practice. In current literature, conventional growth media utilises foetal bovine serum (FBS) [126]. Subsequent FBS-components retained in the stem cells may then induce immune rejection reactions in human recipients [127]. However, human serum (autologous and pooled allogeneic) cannot provide the large outputs required for therapeutic use, as has already been attempted for human mesenchymal stem cells (hMSCs) [128,129]. Instead, research efforts should focus on developing serum-free (‘chemically-defined’) media for ADSCs. For example, Mark *et al*. demonstrated comparable efficacy of hMSC-specific serum-free growth media [126]. However, they did concede that favourable CD105+ expression for cardioregenerative potential was reduced and its chemical composition requires further alterations.

There are strict restrictions on such advanced medicine and therapeutic products (AMTPs) set by regulatory bodies such as the Medicines and Healthcare products Regulatory Agency (MHRA), Federal Drugs Agency (FDA) and Human Tissue Authority (HTA) [130]. For example, in the USA, collagenase use to digest adipose tissue and derive ADSCs results in ‘HCT/P-351’ classification [111]. This is the drug/biologics pathway and requires FDA approval with a high barrier to entry in the clinical market. In addition, there are no standardised protocols for assessing these products and patient safety may be impacted as a result.

The work by Lindegren *et al*., using irradiated human breast tissue and conducting a gene expression analysis, is an important step forward in the field and similar studies should be conducted for corroboration [39]. Fibrotic mechanisms may vary across species and so these results are the most relevant for future clinical practice. Nonetheless, human recruitment in these surgical trials is difficult and only a small sample size was possible. The Royal College of Surgeons of England recently published a study citing service pressures, complicated bureaucracy, and lack of infrastructure (e.g. no trial nurses) as the most common barriers [131]. Gene expression analysis of tissue biopsies can be taken further with whole transcriptome analysis with total RNA sequencing, which will help identify single nucleotide polymorphisms (SNPs) in patients [132]. These slight variations in genetic coding can alter function of proteins involved in pro-/anti-fibrotic pathways, which may enhance or inhibit the efficacy of AFT against RIF of the skin. This form of ‘personalised medicine’ will help to improve individual patient outcomes.

Ultimately, research will continue on discovering the underlying mechanisms by which fibrosis is attenuated or even reversed. The identified factors could be used to enrich fat grafts or even be used as therapeutic options by themselves to entirely bypass the strict regulations currently in place for cellular therapies.

## Limitations

A significant limitation in this field of research is that most studies use varying methodologies, which restricts our ability to compare study results and conduct a statistical meta-analysis. These differences included *in vitro* cell types, *in vivo* animal models, radiation protocols, measured outcomes and their quantification techniques. Although their methods for ADSC-derivation were generally similar, some studies had no mention of how they characterised these stem cells and confirmed their regenerative capacity. A consensus should be reached on key ADSC markers, which will help to standardise future research and increase validity. The same should be done for all factors of study heterogeneity as aforementioned.

## Conclusion

In conclusion, AFT shows promising efficacy in RIF of the skin and likely functions through a combination of immunomodulatory, vasculogenic and anti-fibrotic pathways to reach their clinical effects. Further characterization of these mechanisms will enable identification of potential drug targets, and thereby improve outcomes of future AFT therapies. Future research should explore other cell types within the fat graft such as adipocytes, endothelial cells, stromal cells and their associated progenitor cells.

## Supporting information

S1 Checklist PRISMA checklist. (DOC)

S1 File Risk of bias tables. (PDF)

## Abbreviations

ADSC: autologous-derived stem cell
ADSC-CM: autologous-derived stem cell- conditioned media
AFT: autologous fat transfer
AMPK–AMP: activated protein kinase
AMTPs: advanced medicine and therapeutic products
α-SMA: alpha smooth muscle actin
BAX–Bcl-2: associated X protein
Bcl-2: B cell lymphoma 2
Bid: BH3 interacting-domain death agonist
BMSC: bone marrow derived stem cells
CAL: cell-assisted lipotransfer
COL: collagen
CSF1: colony-stimulating factor 1
CTGF: connective tissue growth factor
CTSF: cathepsin F
DAMP: damage-associated molecular pattern
Dlk: protein delta homolog 1
DMTF1: cyclin-D-binding Myb-like transcription factor 1
ECM: extracellular matrix
ELISA: enzyme-linked immunosorbent assay
FACS: fluorescence-activated cell sorting
FBS: foetal bovine serum
FDA: federal drugs agency
FGF: fibroblast growth factor
FOSL2: FOS-like Antigen 2
GPC3/4: glypican-3/4
GPx: glutathione peroxidase
Gy: Grays
HaCaT: human immortalised keratinocytes
HDLEC: human dermal lymphatic endothelial cells
HDMEC: human microvascular dermal endothelial cell
HFF: human foreskin fibroblasts
HGF: hepatocyte growth factor
hMSCs: human mesenchymal stem cells
HTA: human tissue authority
ICAM-1: intercellular adhesion molecule 1
IF: immunofluorescence
IFN-γ: interferon gamma
IGF: insulin-like growth factor
IHC: immunohistochemistry
IL: interleukin
INHA: inhibin subunit alpha
JNK: c-Jun N-terminal kinases
KGF: keratinocyte growth factor
LC-MS: liquid chromatography
MCP-1: monocyte chemoattractant protein-1
MFAP: microfibrillar-associated protein
MHRA: medicines and healthcare products regulatory agency
MIF: macrophage inhibitory factor
MMP: matrix metalloproteinase
NDST1: N-Deacetylase And N-Sulfotransferase 1
NF-κB: Nuclear factor kappa-light-chain-enhancer of activated B cells
NOK: normal human oral squamous epithelial cells
PDGF: platelet-derived growth factor
PBS: phosphate-buffered saline
PPFIA4: liprin-alpha-4
PRF: platelet-rich fibrin
PRP: platelet-rich plasma
Q-dot: Fluorescence quantum dot
RIF: radiation-induced fibrosis
ROS: reactive oxygen species
RT-qPCR: reverse transcriptase reverse transcription quantitative real-time PCR
Sca1: Stem cells antigen-1
SDF-1: stromal cell-derived factor 1
SNP: single-nucleotide polymorphism
SOD: superoxide dismutase
SVC: stromal vascular cells
SVF: stromal vascular fraction
TGF-β: transforming growth factor beta
Th1: T helper 1 cell
TIMP: tissue inhibitor of metalloproteinases
TMT: tandem mass tag
TNF: tumour necrosis factor
TSG-6: tumour necrosis factor-inducible gene 6 protein
VCAM-1: Vascular cell adhesion protein 1
VEC: vascular endothelial cells
VEGF: vascular endothelial growth factor
vWF: von Willebrand factor
WS1: human skin fibroblast cells
