# Supplementary material for: Exploring the mechanisms behind autologous lipotransfer for radiation-induced fibrosis: A systematic review
Source: PLoS One. 2024 Jan 25;19(1):e0292013. doi: 10.1371/journal.pone.0292013 (PMC10810439; doi:10.1371/journal.pone.0292013)
Supplement: S1 File — (PDF) [file pone.0292013.s002.pdf]

In-vitro studies (OHAT)

Paper	D1	D2	D3	D4	D5	D6	Overall
Haubner et al. 2013	++	−	++	+	−	++	+
Shukla et al.	++	−	++	−	++	++	+
Haubner et al. 2015	++	−	++	+	−	++	+
Yao et al.	++	−	++	++	−	++	+
Yu et al.	++	−	++	++	−	++	+
Ejaz et al.	++	−	++	−	−	++	+
Xiao et al.	++	−	++	++	−	++	+
Saijo et al.	++	−	++	−	−	++	+
Sörgel et al.	++	−	++	++	−	++	+

<u>Legend</u>

Domains

D1: Experimental conditions
D2: Blinding during study
D3: Incomplete data
D4: Exposure characterisation
D5: Outcome assessment
D6: Reporting

Symbols

++ **Definitely low risk of bias**

\+ **Probably low risk of bias**

− **Probably high risk of bias**

-- **Definitely high risk of bias**

In-vivo studies (SYRCLE)

Paper	D1	D2	D3	D4	D5	D6	D7	D8	D9
Borrelli et al. 2020 Nov	−	+	?	?	?	?	+	+	+
Sultan et al.	+	+	?	+	?	+	−	+	+
Forcheron et al.	+	+	?	+	?	?	−	+	+
Riccobono et al. 2012	+	+	?	+	?	?	−	+	+
Garza et al.	−	+	?	?	?	?	+	+	+
Riccobono et al. 2014	+	+	?	+	?	?	−	−	+
Luan et al.	−	+	?	?	?	?	+	+	+
Ejaz et al.	−	+	?	+	?	?	−	+	+
Lindegren et al.	−	+	+	N/A	?	?	+	+	+
Bertrand et al.	+	+	?	+	+	?	+	+	+
Borrelli et al. 2020 Mar	−	−	?	?	?	?	+	+	+
Deleon et al.	−	+	?	+	?	?	+	+	+
Yao et al.	+	+	?	?	?	?	+	+	+
Khademi et al.	−	+	?	?	?	?	−	+	+
Riccobono et al. 2018	+	−	?	?	?	?	−	+	+

**Riccobono et al. 2016**	+	+	?	+	?	?	+	+	+
**Sun et al.**	+	+	?	+	?	?	+	+	+
**Huang et al.**	+	+	?	+	?	?	+	+	+
**Chen et al.**	+	+	?	?	?	?	−	+	+
**Evin et al.**	+	+	?	?	?	?	+	+	+
**Yu et al.**	+	+	?	?	?	?	−	+	+
**Abbas et al.**	+	+	?	?	?	?	+	+	+
**Kim et al.**	+	+	?	?	?	?	+	+	+
**Adem et al.**	+	+	?	+	+	?	+	+	+
**Sowa et al.**	+	+	?	?	?	?	+	+	+

<u>Legend</u>

Domains

D1: Selection bias (sequence generation)
D2: Selection bias (baseline characteristics)
D3: Selection bias (allocation concealment)
D4: Performance bias (random housing)
D5: Performance bias (blinding)
D6: Detection bias (random outcome assessment)
D7: Detection bias (blinding)
D8: Attrition bias (incomplete outcome data)
D9: Reporting bias (selective outcome reporting)

Symbols

+ **Low risk of bias**

− **High risk of bias**

? **Insufficient detail reported to assess risk of bias**

N/A **Not applicable**
